# Localized Intratumoral Ammonium Hydroxide Administration Demonstrates Changes in Tumor Architecture and Renal Response in a Murine Breast Cancer Xenograft Model

**DOI:** 10.3390/cimb48080803

**Published:** 2026-08-07

**Authors:** Hemalata Deshmukh, Camille Schacherer, Kyunghoon Yeom, Alaina Rivera, Yusuff Olayiwola, Lauren Gollahon

**Affiliations:** Department of Biological Sciences, Texas Tech University, 2500 Broadway, Lubbock, TX 79409, USA; hemalata.deshmukh@ttu.edu (H.D.); camschac@ttu.edu (C.S.); kyyeom@ttu.edu (K.Y.); alairive@ttu.edu (A.R.); yolayiwo@ttu.edu (Y.O.)

**Keywords:** breast cancer, ammonium hydroxide, intratumoral therapy, MDA-MB-231, apoptosis, inflammation, Ki-67, ammonia transporters, RHBG, RHCG, renal safety

## Abstract

Breast cancer remains a leading cause of cancer-related mortality worldwide, highlighting the need for novel therapeutic strategies that selectively target tumor cells while minimizing systemic toxicity. Dietary ammonium hydroxide enhancement (AHE) has previously been shown to modulate metabolic pathways in animal studies. However, its potential as a localized anticancer therapy has not been investigated. In the present study, we evaluated the antitumor efficacy and systemic safety of NH_4_OH using complementary in vitro and orthotopic breast cancer xenograft models. MDA-MB-231 breast cancer cells and non-tumorigenic MCF10A mammary epithelial cells were treated with increasing concentrations of NH_4_OH (2.5–225 µM) to assess dose-dependent effects on cell proliferation, viability, and apoptosis. Following this, MDA-MB-231 cells were orthotopically xenografted into female athymic nude mice and treated by intratumoral injection with NH_4_OH using a stepwise dose-escalation regimen (0.01%, 0.1%, and 0.5%; total volume of 20 μL per tumor divided between two injection sites) or phosphate-buffered saline (PBS). Differences between treatment groups of mammary tumors and kidney tissues were analyzed molecularly and histologically. NH_4_OH significantly suppressed MDA-MB-231 cell growth and metabolic activity, with minimal effects on MCF10A cells, and induced apoptosis in MDA-MB-231 cells without detectable apoptotic induction in MCF10A cells. In vivo, although tumor volume only showed a non-significant downward trend, histological and molecular analyses demonstrated substantial alterations in tumor biology. NH_4_OH treatment induced molecular changes consistent with an antitumor response, including increased Caspase-3 and p53 expression, reduced BCL2 and Ki-67 expression, and attenuation of TNFα, IL-6, and TLR4 inflammatory signaling. Furthermore, the tumor architecture in T-NH tumors displayed increased pale eosinophilic regions and reduced cellular density, suggestive of treatment-associated tumor tissue disruption. Histological analysis of kidney tissue showed no evidence of overt renal toxicity. Indeed, localized NH_4_OH administration was associated with reduced renal inflammatory and apoptotic signaling, preserved renal morphology, and increased expression of the ammonia transporters RHBG and RHCG. Cross-sectional morphometric measurements showed decreased area for the distal convoluted tubules in NH_4_OH-treated samples. Although this initial preclinical study was limited by a relatively small sample size, further studies are warranted to validate these findings and define the molecular mechanisms underlying NH_4_OH-mediated antitumor activity. Collectively, these findings suggest that intratumoral NH_4_OH modulates tumor metabolic, inflammatory, and apoptotic pathways associated with a less aggressive tumor phenotype while showing no overt molecular or histological evidence of renal injury, supporting further investigation as a localized metabolic intervention targeting molecular and histological drivers of breast cancer progression.

## 1. Introduction

Breast cancer remains one of the most frequently diagnosed malignancies and a leading cause of cancer-related mortality worldwide [1,2,3,4]. Despite major advances in all treatment strategies, tumor recurrence, metastasis, and therapeutic resistance continue to limit long-term clinical outcomes [5,6,7,8]. These challenges are particularly evident in aggressive breast cancer subtypes, such as triple-negative breast cancer [9], where fewer targeted treatment options are available [10,11]. Therefore, innovative therapeutic strategies that suppress tumor growth while minimizing systemic toxicity are urgently needed [12,13,14].

Cancer progression is strongly influenced by metabolic reprogramming within the tumor microenvironment [15,16,17]. Tumor cells adapt their metabolism to support rapid proliferation, survival under stress, and resistance to cell death [18,19,20]. Among these metabolic adaptations, altered glutamine and nitrogen metabolism are especially important, as glutamine provides carbon and nitrogen for biosynthesis, redox balance, and energy production. Ammonia is a major by-product of glutamine metabolism and has emerged as a biologically active regulator within tumors [21,22]. Ammonia has a context-dependent role in cancer metabolism. At physiological or moderate levels, ammonia generated from glutamine metabolism may support tumor adaptation and biosynthetic signaling [21]. However, excessive ammonia accumulation can overwhelm cellular homeostasis, induce metabolic stress, and promote cell death [16]. Thus, ammonia metabolism may represent a therapeutically exploitable vulnerability in cancer [21,23].

Our previous studies using ammonium hydroxide-enhanced (AHE) dietary protein in a diet-induced obese mouse model demonstrated that controlled modulation of ammonium availability can influence metabolic and molecular pathways relevant to cancer and systemic physiology [24,25]. In C3H/HeJ mice fed high-fat diets supplemented with AHE, we observed delayed tumor onset, improved survival, and changes in gut microbiota, adipose tissue signaling and secretory products, β-catenin signaling, glutamine synthetase expression, and amino acid-related metabolic pathways [26,27,28]. These findings suggest that ammonium-based interventions can influence cellular metabolism in a tissue-dependent manner and provide a rationale for investigating whether ammonium hydroxide can be used to alter tumor biology directly [29].

Intratumoral injection therapy offers a strategy to deliver treatment directly into the tumor mass, allowing high local exposure while potentially limiting systemic toxicity [15,30,31]. This approach is particularly attractive for agents that modify the tumor microenvironment or disrupt tumor metabolism [32]. Building on our previous AHE findings, we evaluated ammonium hydroxide (NH_4_OH) as a potential intratumoral therapy for breast cancer [26,28,29]. We hypothesized that localized NH_4_OH delivery would suppress tumor-promoting pathways, induce apoptotic signaling, reduce proliferative activity, and minimize systemic toxicity.

In the present study, we examined the effects of NH_4_OH using complementary in vitro and in vivo breast cancer models. MDA-MB-231 breast cancer cells and MCF10A non-tumorigenic mammary epithelial cells were used to assess cell growth, XTT absorbance, and apoptosis following NH_4_OH exposure. In vivo, MDA-MB-231 cells were used to establish xenograft breast tumors in female athymic nude mice, followed by direct intratumoral injection of NH_4_OH or PBS. Tumor tissues were evaluated for inflammatory signaling, apoptotic regulators, proliferative activity, and histological morphology. Because local treatment may have systemic consequences, kidney tissues were also analyzed for histomorphological changes, inflammatory markers, apoptotic signaling, and ammonia transporter expression. This pilot study provides a framework for evaluating NH_4_OH as a localized ammonia-based metabolic intervention for breast cancer while assessing potential systemic renal effects.

## 2. Materials and Methods

### 2.1. Determination and Preparation of Ammonium Hydroxide Treatment Concentrations

This is the first reported study for using Ammonium hydroxide (NH_4_OH), was a cancer therapy through intratumoral injection. Therefore, no previously established dosing regimen was available. Concentration selection was guided by our laboratory’s previous studies demonstrating that ammonium hydroxide enhancement (AHE) modulates metabolic pathways and improves physiological outcomes without evidence of overt systemic toxicity in vivo [26]. These studies established the biological feasibility of controlled ammonium hydroxide exposure and provided the rationale for evaluating NH_4_OH as a localized therapeutic agent in breast cancer [24,25,27,28,29]. Ammonium hydroxide (NH_4_OH), extra pure, 10 wt.% in water (Cat. No. 458680010; Thermo Fisher Scientific, Waltham, MA, USA), was used to prepare all treatment solutions.

For in vitro experiments, NH_4_OH was added to complete culture medium to obtain final concentrations of 2.5, 5.25, 11, 22.5, 45, and 225 μM. This concentration range was selected to evaluate cellular responsiveness to NH_4_OH.

Calculations for determining these concentrations was as follows:

The labeled 10 wt% NH_4_OH stock was operationally treated as:

10%=100 g/L

Using the molecular weight of NH_4_OH, 35.05 g/mol:

$$\frac{100\;g/L}{35.05\;g/\mathrm{mol}}=2.85\;\mathrm{mol}/L$$

Therefore, the nominal stock concentration was approximately 2.85 M NH_4_OH.

PubChem lists the molecular weight of NH_4_OH as 35.046 g/mol.

The 225 mM intermediate stock was prepared as:

$$C_{2}=\frac{2.85\;M\times 0.790\;\mathrm{mL}}{10\;\mathrm{mL}}=0.225\;M$$

The 225 µM working stock was then prepared by a 1:1000 dilution:

$$225\;\mathrm{mM}\times \frac{0.100\;\mathrm{mL}}{100\;\mathrm{mL}}=0.225\;\mathrm{mM}=225\;\mu M$$

Initial screening concentrations were 225, 45, 22.5, 11.25, 5.625, and 2.81 µM. Based on the screening results, 45 and 2.5 µM were selected for subsequent experiments.

Cell culture medium pH (approximately 8.3–9.7) was measured only to characterize the physicochemical environment following NH_4_OH addition and was not used to determine the treatment concentrations. Initial screening using the XTT cell viability assay demonstrated that NH_4_OH reduced MDA-MB-231 cell viability across the tested concentrations. Based on these findings, 2.5 and 45 μM were selected for subsequent growth curve and Annexin V/PI apoptosis assays, representing low and biologically active treatment concentrations while minimizing nonspecific cytotoxicity. These in vitro concentrations were selected independently and were not intended to directly correspond to the intratumoral concentrations used in vivo.

### 2.2. Cell Culture

The human triple-negative breast cancer cell line MDA-MB-231 (Cat. No. HTB-26; American Type Culture Collection (ATCC), Manassas, VA, USA) and the non-tumorigenic human mammary epithelial cell line MCF10A (Cat. No. CRL-10317; ATCC, Manassas, VA, USA) were used in this study [33]. MDA-MB-231 cells were cultured in high-glucose Dulbecco’s Modified Eagle’s Medium (DMEM; Gibco, Thermo Fisher Scientific, Waltham, MA, USA) supplemented with 10% fetal bovine serum (FBS; Cat. No. F-0500-D; Atlas Biologicals, Fort Collins, CO, USA) and 1% penicillin/streptomycin (Cat. No. 15140-122; Gibco, Thermo Fisher Scientific, Waltham, MA, USA). MCF10A cells were cultured in RPMI-1640 medium supplemented with 10% FBS, 1% L-glutamine, and 1% penicillin/streptomycin. Cells were maintained at 37 °C in a humidified incubator containing 5% CO_2_. Culture medium was replaced every 2–3 days, and cells were passaged at 70–80% confluence using standard trypsinization procedures.

### 2.3. In Vitro Assays

#### 2.3.1. Growth Curve Assays

The effect of ammonium hydroxide (NH_4_OH) on cell proliferation was assessed using a growth curve assay. MDA-MB-231 and MCF10A cells were seeded at a density of 5 × 10^4^ cells/well in 48-well tissue culture-treated Nunclon™ Delta surface plates (Cat. No. 150687; Thermo Fisher Scientific, Waltham, MA, USA) containing complete growth medium and allowed to attach overnight at 37 °C in a humidified incubator with 5% CO_2_. Cells were then treated with NH_4_OH at concentrations of 2.5 µM or 45 µM, while untreated cells served as controls. Cell proliferation was monitored from day 0 to day 6. At each time point, cells were trypsinized using 0.25% Trypsin-EDTA (Cat. No. 25200-056; Gibco, Thermo Fisher Scientific, Waltham, MA, USA), collected, and viable cells were counted using trypan blue exclusion (Cat. No. 1691049; MP Biomedicals, Solon, OH, USA) and a hemocytometer (Reichert Scientific Instruments, Buffalo, NY, USA). All experiments were performed in triplicate, and growth curves were generated by plotting the mean viable cell number over time.

#### 2.3.2. XTT Cell Viability Assay

Cellular metabolic activity/viability was evaluated using the XTT Cell Viability Kit (Cat. No. 30007; Biotium, Fremont, CA, USA). MDA-MB-231 and MCF10A cells were seeded at a density of 5 × 10^3^ cells/well in 96-well plates and allowed to attach overnight under standard culture conditions. The following day, cells were treated with NH_4_OH at concentrations of 2.5, 5.6, 11, 22.5, 45, or 225 µM, corresponding to pH values of 8.3, 8.6, 8.8, 9.1, 9.3, and 9.7, respectively (data shown is with high and low concentrations only). Untreated cells served as controls. After 1, 3, or 48 h of treatment, XTT reagent was added according to the manufacturer’s instructions, and plates were incubated for 2–4 h to allow color development. Absorbance was measured at 475 nm using a microplate reader Synergy™ H1 Hybrid Multi-Mode Microplate Reader (BioTek Instruments, Winooski, VT, USA). Data acquisition and analysis were performed using Gen5™ Microplate Reader and Imaging Software (Version 3.06, BioTek Instruments, Winooski, VT, USA). Experiments were performed in triplicate.

#### 2.3.3. Annexin V/PI Apoptosis Assay

Apoptosis was assessed using Annexin V/propidium iodide (PI) staining followed by flow cytometry. MDA-MB-231 and MCF10A cells were seeded at a density of 1 × 10^5^ cells/well in 6-well plates and allowed to attach overnight under standard culture conditions. Cells were then treated with NH_4_OH at 2.5 µM or 45 µM for 3, 12, or 24 h, while untreated cells served as controls. Following treatment, cells were harvested, washed with cold PBS, and stained using the Annexin V-FITC Apoptosis Detection Kit (Cat. No. BMS500FI-300; eBioscience™, Invitrogen, Thermo Fisher Scientific, Waltham, MA, USA) according to the manufacturer’s instructions. Stained cells were analyzed using a BD FACSAria™ flow cytometer (BD Biosciences, San Jose, CA, USA), and data acquisition and analysis were performed using FACSDiva software (Version 6.1.3; BD Biosciences, San Jose, CA, USA). Cell populations were classified as live, early apoptotic, late apoptotic, or naked nuclei based on Annexin V-FITC/PI staining. The 12 h and 24 h data are presented in the main manuscript, whereas the 3 h data are provided in Supplementary Figure S9. Experiments were performed in triplicate.

### 2.4. Animals and Experimental Design

All animal procedures were approved by the Institutional Animal Care and Use Committee (IACUC) of Texas Tech University (Protocol No. 2025-1621 approved on 23 June 2025) and conducted in accordance with institutional guidelines for the care and use of laboratory animals. Female athymic nude mice (nu/nu; 6–8 weeks of age) were obtained from Charles River Laboratories (Crl:NU-Foxn1^nu; strain code 088, Wilmington, MA, USA) and housed under specific pathogen-free conditions with ad libitum access to food and water under a 12-h light/dark cycle. Animals were acclimated for 1 week. Twenty mice were randomly assigned to four experimental groups (*n* = 5 per group): (i) tumor-bearing mice receiving intratumoral phosphate-buffered saline (T-PBS), (ii) tumor-bearing mice receiving intratumoral ammonium hydroxide (T-NH), (iii) non-tumor-bearing mice receiving subcutaneous PBS (PBS), and (iv) non-tumor-bearing mice receiving subcutaneous ammonium hydroxide (NH). Although five mice were initially assigned to each tumor-bearing group, successful tumor engraftment was achieved in four of the five mice in the T-PBS group, whereas all five mice in the T-NH group developed tumors. Therefore, analyses of tumor-bearing animals were performed using T-PBS (*n* = 4) and T-NH (*n* = 5).

### 2.5. Orthotopic Breast Cancer Xenograft Model

Orthotopic breast tumors were established using the human triple-negative breast cancer cell line MDA-MB-231 as previously described, with minor modifications [34]. Mice were anesthetized with inhaled isoflurane, and bilateral injections were performed into the inguinal mammary fat pads. Each mouse received a total of 5 × 10^6^ cells (2.5 × 10^6^ cells suspended in 25 µL sterile PBS per mammary fat pad) using a 30-gauge needle.

### 2.6. Intratumoral NH_4_OH Treatment

Tumor growth was monitored two to three times per week using digital Vernier calipers. Once tumors reached a volume of approximately 50–100 mm^3^, treatment was initiated. For in vivo studies, NH_4_OH was diluted in sterile phosphate-buffered saline (PBS) to prepare treatment concentrations of 0.01%, 0.1%, and 0.5%. All treated animals received the same stepwise dose-escalation regimen. Because no previously established intratumoral NH_4_OH dosing protocol was available, these concentrations were selected independently of the in vitro concentrations to improve local tolerability while evaluating biological activity following localized administration. Consequently, no direct conversion between the in vitro and in vivo concentrations was performed. Fresh NH_4_OH solutions were prepared immediately before each experiment.

All tumor-bearing mice assigned to the NH_4_OH treatment group received the same stepwise dose-escalation regimen consisting of 0.01%, 0.1%, and 0.5% NH_4_OH administered at weekly intervals. This strategy was selected because no previously established dosing regimen for intratumoral ammonium hydroxide was available and was intended to improve local tolerability while progressively evaluating biological activity. Table 1 summarizes the dose calculations and their relationships between in vitro and in vivo transitions.

Calculations for in vivo percentages converted to nominal molarity were as follows:

Because each solution was prepared directly from the same 10 wt% stock:

0.01% formulation

$$2.85\;M\times \frac{10\;\mu L}{10,000\;\mu L}=0.00285\;M=2.85\;\mathrm{mM}$$

Amount administered in 20 µL:

$$2.85\;\mathrm{mmol}/L\times 20\times 10^{-6}\;L=57\;\mathrm{nmol}/\mathrm{tumor}$$

0.1% formulation

$$2.85\;M\times \frac{100}{10,000}=0.0285\;M=28.5\;\mathrm{mM}$$

Amount in 20 µL:

$$28.5\;\mathrm{mmol}/L\times 20\times 10^{-6}\;L=570\;\mathrm{nmol}/\mathrm{tumor}$$

0.5% formulation

$$2.85\;M\times \frac{500}{10,000}=0.1425\;M=142.5\;\mathrm{mM}$$

Amount in 20 µL:

$$142.5\;\mathrm{mmol}/L\times 20\times 10^{-6}\;L=2.85\;\mu \mathrm{mol}/\mathrm{tumor}$$

Thus, the nominal concentrations in the syringe were higher than the active in vitro concentrations. The in vivo material was injected into a heterogeneous, buffered solid tumor, where it would undergo local dilution, diffusion, protonation, vascular clearance, and neutralization. The actual concentration experienced by individual tumor cells was not measured.

A total volume of 20 µL was administered per tumor using two injection sites to ensure uniform intratumoral distribution. These sites differed for each treatment. Treatments were administered once or twice weekly, with no more than two injections per week. Tumor-bearing control mice received an equivalent volume of PBS following the same injection schedule. Non-tumor-bearing control animals received subcutaneous injections of either NH_4_OH or PBS (10 µL per mammary fat pad; total volume 20 µL) using a 31-gauge needle.

### 2.7. Tumor Monitoring and Tissue Collection

Tumor dimensions were measured two to three times weekly, and tumor volume was calculated using the formula: Tumor volume (mm^3^) = (length × width^2^)/2. Animals were monitored daily for body condition and signs of distress according to IACUC-approved humane endpoint criteria. At the study endpoint, mice were euthanized by isoflurane overdose followed by thoracotomy as a secondary method to ensure death. Tumor and kidney tissues were harvested, with portions either snap-frozen in liquid nitrogen for molecular analyses or fixed in 10% neutral-buffered formalin for histological and immunohistochemical evaluation.

### 2.8. Quantitative Real-Time PCR (qRT-PCR)

Total RNA was isolated from frozen tumor and kidney tissues using RNAzol RT reagent (Cat. No. RN190; Molecular Research Center Inc., Cincinnati, OH, USA) according to the manufacturer’s instructions. RNA concentration and purity were determined using a NanoDrop One spectrophotometer (Thermo Fisher Scientific, Waltham, MA, USA). Complementary DNA (cDNA) was synthesized from total RNA using the Maxima First Strand cDNA Synthesis Kit with dsDNase (Cat. No. K1671; Thermo Fisher Scientific, Waltham, MA, USA) and a C1000 Touch™ Thermal Cycler (Bio-Rad Laboratories, Hercules, CA, USA).

Quantitative real-time PCR was performed using a CFX96 Touch™ Deep Well Real-Time PCR Detection System (Bio-Rad Laboratories, Hercules, CA, USA) with PowerUp™ SYBR™ Green Master Mix (Applied Biosystems, Thermo Fisher Scientific, Waltham, MA, USA). Gene-specific primers were used to evaluate ammonia transporters (*RHBG* and *RHCG*), and inflammatory markers (*TNFα*, *IL-1β*, *TLR4*, and *NF-κB*). *β-Actin* served as the endogenous reference gene. Relative gene expression was calculated using the 2^−ΔΔCt^ method and expressed as fold change relative to the appropriate control group [35]. Primer sequences are listed in Table 2.

### 2.9. Western Blot Analysis

Total protein was extracted from frozen tumor and kidney tissues using radioimmunoprecipitation assay (RIPA) buffer supplemented with protease and phosphatase inhibitor cocktails. Tissue samples were homogenized on ice using a bead mill homogenizer, followed by centrifugation to collect the protein-containing supernatant. Protein concentrations were determined using a bicinchoninic acid (BCA) protein assay kit (Cat. No. 23227; Thermo Fisher Scientific, Rockford, IL, USA). Equal amounts of protein (40 μg) were separated by SDS-PAGE and transferred onto polyvinylidene difluoride (PVDF) membranes (Cat. No. 1704273; Bio-Rad Laboratories, Hercules, CA, USA). Membranes were blocked with 5% bovine serum albumin (BSA) for 1 h at room temperature and incubated overnight at 4 °C with primary antibodies against TNFα, IL-6, TLR4, NF-κB, Caspase-3, p53, BCL2, RHBG, and RHCG. After incubation with horseradish peroxidase (HRP)-conjugated secondary antibodies, protein bands were detected using SuperSignal™ West Pico PLUS Chemiluminescent Substrate (Cat. No. 34580; Thermo Fisher Scientific, Waltham, MA, USA).

Images were acquired using an LI-COR Odyssey Fc Imaging System (Model 2800; LI-COR Biosciences, Lincoln, NE, USA) and analyzed using Image Studio software (Version 4.0; LI-COR Biosciences, Lincoln, NE, USA). Band intensities were quantified using ImageJ software (Version 1.54f; Wayne Rasband and contributors, National Institutes of Health, Bethesda, MD, USA) and normalized to β-actin. Representative immunoblots are shown, and densitometric analysis was performed using all available biological samples. Primary antibodies and working dilutions are provided in Table 3.

### 2.10. Histology and Immunohistochemistry

Tumor and kidney tissues were fixed in 10% neutral-buffered formalin (Cat. No. 232166; StatLab Medical Products, McKinney, TX, USA), routinely processed, and embedded in paraffin using a Leica EG1160 tissue embedding station (Leica, Nussloch, Germany). Serial sections (5 µm) were prepared using a microtome Leica RM2025 rotary microtome (Leica Biosystems, Wetzlar, Germany) and placed on to a charged glass microscope slide (Cat. No. 1358W; Globe Scientific Inc., Mahwah, NJ, USA)

For histological evaluation, sections were stained with hematoxylin and eosin (H&E) following standard procedures [36]. Briefly, paraffin sections were deparaffinized in xylene (Cat. No. 8400-1; Thermo Fisher Scientific, Waltham, MA, USA), rehydrated through graded ethanol solutions, stained with Mayer’s hematoxylin (Cat. No. 26043-05, Electron Microscopy Sciences, Hatfield, PA, USA), differentiated, blued, counterstained with eosin Y (Cat. No. 2850-32, Thermo Fisher Scientific, Waltham, MA, USA), dehydrated, cleared, and mounted with coverslips. Images were acquired using bright-field microscopy Olympus BX51 microscope (Olympus, Tokyo, Japan) and an EVOS XL Core Imaging System (Thermo Fisher Scientific, Waltham, MA, USA). Scale bars were added using ImageJ software (Version 1.54f; Wayne Rasband and contributors, National Institutes of Health, Bethesda, MD, USA). Kidney morphometric analysis was performed using QuPath software (version 0.7.0; University of Edinburgh, Edinburgh, UK) to quantify proximal tubular area, distal tubular area, glomerular area, and Bowman’s space area. Measurements were obtained from multiple representative fields per section and averaged for each animal prior to statistical analysis.

Immunohistochemical (IHC) staining was performed using the Mouse and Rabbit Specific HRP/DAB (ABC) Detection IHC Kit (Cat. No. ab64264; Abcam, Cambridge, UK) according to the manufacturer’s instructions. Briefly for IHC, paraffin sections were deparaffinized and rehydrated. Endogenous peroxidase activity was blocked by incubating sections in 3% hydrogen peroxide at room temperature. Slides were subjected to heat-induced antigen retrieval using citrate buffer (pH 6.0) and incubated overnight at 60 °C. Non-specific background staining was blocked using a protein block. Sections were then incubated with the primary antibodies against Caspase-3, p53, BCL2, and Ki-67 for 1 h at room temperature. Sections were incubated with a Biotinylated Goat Anti-Polyvalent secondary antibody (provided with a kit and incubated with Streptavidin Peroxidase. Immunoreactivity was visualized using 3,3′-diaminobenzidine (DAB) as the chromogen, and counterstained with Mayer’s hematoxylin. Slides were dehydrated, and coverslips were mounted using CytoSeal (Cat. No. 8310-4; Epredia, Kalamazoo, MI, USA) in preparation for examination under light microscopy. Representative images were acquired under identical microscope settings for qualitative comparison among experimental groups. Images were acquired using an EVOS XL Core Imaging System (Thermo Fisher Scientific, Waltham, MA, USA). Scale bars were added using ImageJ software (Version 1.54f; Wayne Rasband and contributors, National Institutes of Health, Bethesda, MD, USA).

For Qupath analysis, histological structures were annotated manually using the polygon annotation tool in QuPath based on histological criteria. Glomeruli were classified by their rounded shape and tufts of capillaries enclosed structure within Bowman’s capsule. Bowman’s space was defined as the clear crescent-shaped space between the glomerular tuft and the layer of Bowman’s capsule. Proximal convoluted tubules were distinguished by the larger, irregular, brush border lumen; distal convoluted tubules were identified by smaller, rounder shape with clearer white regular lumen.

For quantitative analysis of IHC staining, whole-slide digital images of each IHC-stained tumor section were imported into QuPath. Between one and three manually drawn polygon regions of interest (ROIs) were generated per section, depending on the distribution and extent of viable tumor tissue. ROIs were manually annotated using the polygon annotation tool to include viable tumor regions containing morphologically identifiable tumor cells with visible nuclear staining while excluding necrotic areas, tissue folds, edge artifacts, staining artifacts, and adjacent non-tumor tissue. Positive nuclear staining was quantified within the annotated ROIs using the Positive Cell Detection tool in QuPath. The percentage of DAB-positive nuclei was calculated as the number of DAB-positive nuclei divided by the total number of detected nuclei within each annotated ROI. The same detection parameters were applied consistently across all markers and images: detection image, hematoxylin OD; requested pixel size, 2.0 µm; background radius, 15 µm; score compartment, nucleus: DAB OD mean; and positivity threshold, 0.4. An example workflow output is provided in Supplementary Figure S1.

### 2.11. Statistical Analysis

All statistical analyses were performed using GraphPad Prism version 10.0 (GraphPad Software, San Diego, CA, USA). Data are presented as mean ± standard error of the mean (SEM). In vitro experiments were performed in triplicate. For in vivo tumor-bearing groups, T-PBS included four animals and T-NH included five animals. Comparisons between two groups were performed using an Unpaired *t*-test with Welch’s correction. Comparisons among multiple groups were analyzed using one-way analysis of variance (ANOVA) followed by Fisher’s least significant difference (LSD) post hoc test. For Western blot analyses, protein expression was quantified using all animals in each group, while representative blots show two samples per group. Differences were considered statistically significant at * *p* < 0.05, unless otherwise mentioned.

## 3. Results

### 3.1. In Vitro Analyses

#### 3.1.1. NH_4_OH Suppresses MDA-MB-231 Breast Cancer Cell Growth and Metabolic Activity In Vitro

**Figure 1:**
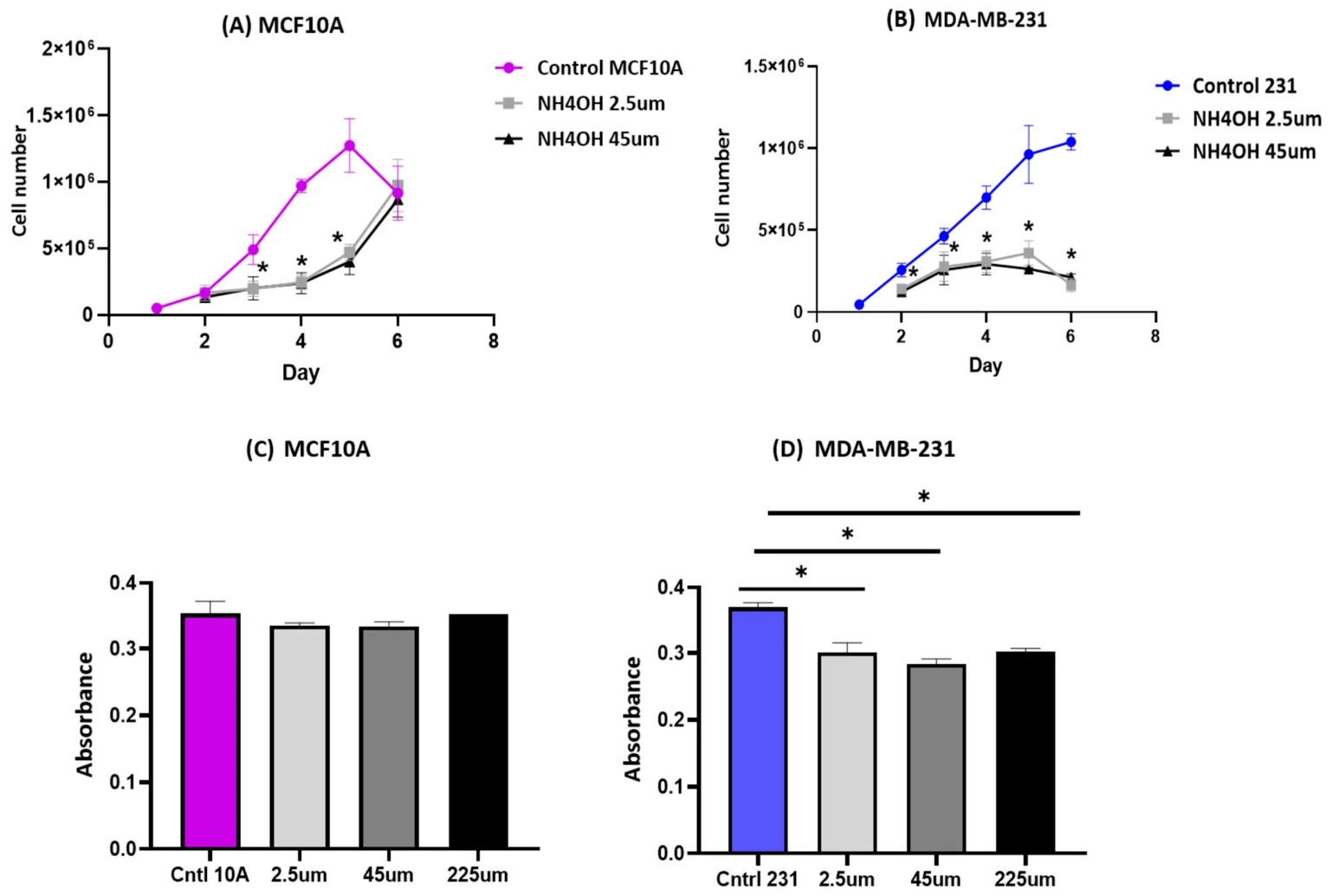
Growth curve and XTT analysis of NH_4_OH-treated MCF10A and MDA-MB-231 cells. MCF10A and MDA-MB-231 cells were treated with NH_4_OH and assessed by growth curve analysis and XTT assay. (**A**) MCF10A and (**B**) MDA-MB-231 cell growth following treatment with 2.5 µM or 45 µM NH_4_OH from day 0 to day 6. (**C**) MCF10A and (**D**) MDA-MB-231 XTT absorbance following treatment with 2.5 µM, 45 µM or 225 µM NH_4_OH. Data are presented as mean ± SEM. * *p* < 0.05 versus control.

To evaluate the effect of NH_4_OH on breast cancer cell growth and cellular metabolic activity, MDA-MB-231 breast cancer cells and MCF10A non-tumorigenic mammary epithelial cells were treated with 2.5 µM or 45 µM NH_4_OH and assessed using growth curve analysis and XTT assay. In MCF10A cells, untreated control cells showed progressive growth over time, while NH_4_OH treatment reduced cell numbers at selected intermediate time points (Figure 1A). However, by the later time point, cell numbers in NH_4_OH-treated MCF10A cells approached those of untreated control cells, suggesting that the growth-inhibitory effect in non-tumorigenic mammary epithelial cells was limited and transient. In contrast, MDA-MB-231 cells demonstrated sustained growth suppression following NH_4_OH exposure (Figure 1B). Untreated control MDA-MB-231 cells continued to increase over the experimental period, whereas both 2.5 µM and 45 µM NH_4_OH markedly attenuated the increase in cell number. Significant reductions were observed at the indicated time points, indicating that NH_4_OH suppresses MDA-MB-231 breast cancer cell growth in vitro. XTT assay further demonstrated differential responses between the two cell lines (Figure 1C,D). In MCF10A cells, NH_4_OH treatment produced only minimal changes in XTT absorbance compared with untreated control cells (Figure 1C), suggesting limited effects on metabolic activity/viability in non-tumorigenic mammary epithelial cells under these conditions. In contrast, NH_4_OH treatment significantly decreased XTT absorbance in MDA-MB-231 cells at all tested concentrations (2.5, 45, and 225 µM) compared with untreated control cells (Figure 1D). Although all three concentrations significantly reduced metabolic activity, the response was not strictly concentration-dependent, as the greatest reduction was observed at 45 µM rather than at the highest concentration tested (225 µM), Figures S7, S8 and S10.

#### 3.1.2. NH_4_OH Treatment Induces Apoptosis in MDA-MB-231 Cells

**Figure 2:**
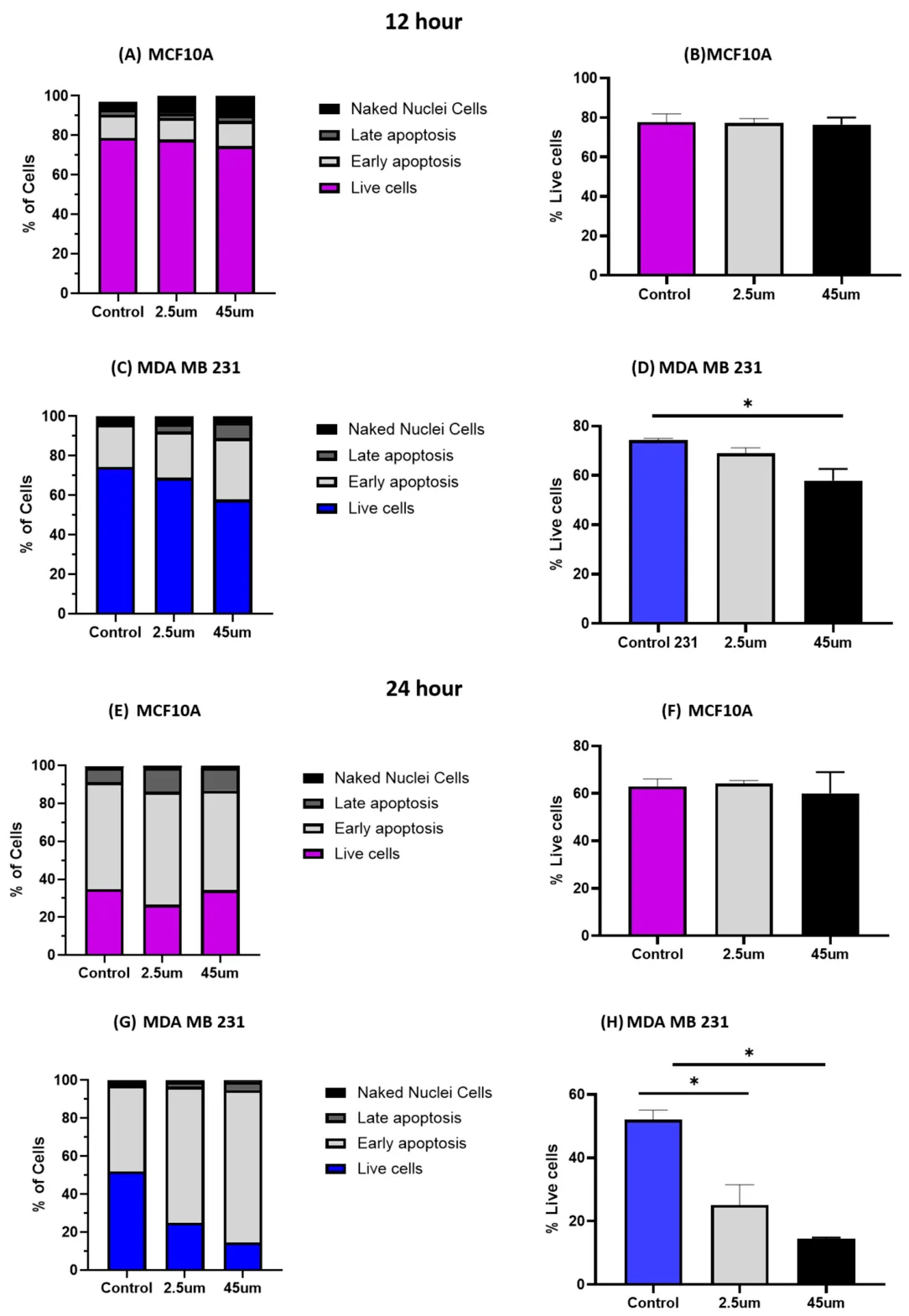
Annexin V-FITC/PI analysis of NH_4_OH-treated MCF10A and MDA-MB-231 cells. MCF10A and MDA-MB-231 cells were treated with NH_4_OH (2.5 or 45 µM) for 12 h (**A**–**D**) or 24 h (**E**–**H**) and analyzed by Annexin V-FITC/PI flow cytometry. Stacked bar graphs show the percentages of live cells, early apoptotic cells, late apoptotic cells, and naked nuclei in MCF10A (**A**,**E**) and MDA-MB-231 (**C**,**G**) cells. Quantification of the percentage of live cells is shown for MCF10A (**B**,**F**) and MDA-MB-231 (**D**,**H**). Data are presented as mean ± SEM. Statistical significance was determined using two-way ANOVA followed by Fisher’s least significant difference (LSD) multiple-comparisons test. * *p* < 0.05.

To determine whether NH_4_OH-mediated growth suppression was associated with apoptosis, MCF10A and MDA-MB-231 cells were treated with NH_4_OH and analyzed by Annexin V-FITC/PI flow cytometry after 12 h and 24 h of treatment. The distributions of live cells, early apoptotic cells, late apoptotic cells, and naked nuclei are shown in Figure 2A,C,E,G, while quantification of the percentage of live cells is presented in Figure 2B,D,F,H. At 12 h, MCF10A cells remained predominantly viable across all treatment groups, with minimal changes in apoptotic cell populations or the percentage of live cells (Figure 2A,B). In contrast, MDA-MB-231 cells exhibited a reduction in the percentage of live cells following NH_4_OH treatment, reaching statistical significance at 45 µM (Figure 2C,D). Following 24 h of treatment, MCF10A cells displayed a lower baseline percentage of live cells than at 12 h; however, NH_4_OH treatment did not further reduce cell viability or substantially alter apoptotic cell populations compared with the time-matched control (Figure 2E,F and Table S1). Conversely, MDA-MB-231 cells demonstrated a marked reduction in the percentage of live cells together with increased apoptotic cell populations following NH_4_OH treatment, with significant decreases in live-cell percentages observed at both 2.5 µM and 45 µM compared with untreated controls (Figure 2G,H). These findings demonstrate that NH_4_OH preferentially induces apoptosis in MDA-MB-231 breast cancer cells while exerting minimal effects on the viability of non-tumorigenic MCF10A cells.

### 3.2. In Vivo Mammary Tumor Analyses of Intratumoral NH_4_OH Treatment

#### 3.2.1. Analysis of Mammary Tumor Development and Histological Features from an MDA-MB-231 Xenograft Model

**Figure 3:**
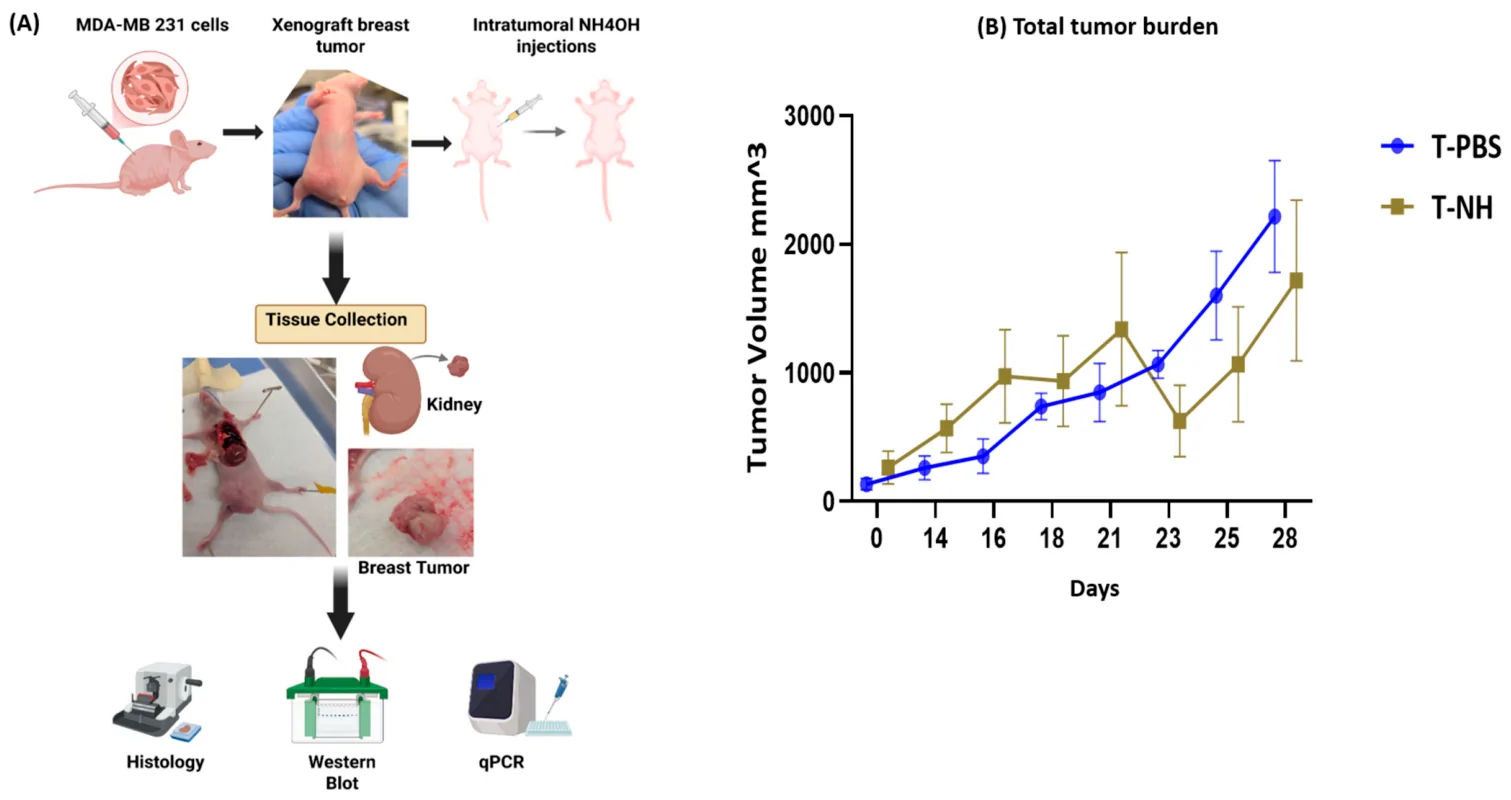
In vivo experimental overview and tumor burden following intratumoral NH_4_OH treatment. (**A**) Schematic overview of the xenograft study design. MDA-MB-231 cells were injected into the inguinal mammary fat pads of female athymic nude mice to establish breast tumors. Once tumors reached 50–100 mm^3^, mice received intratumoral injections of NH_4_OH or PBS control. Tumor and kidney tissues were collected at endpoint for histological, Western blot, and qPCR analyses. (**B**) Total tumor burden in T-PBS and T-NH groups over the study period. NH_4_OH-treated tumors showed a non-significant trend toward reduced tumor burden compared with PBS-treated controls. Data are presented as mean ± SEM and were analyzed using two-way repeated-measures ANOVA followed by Šídák’s multiple-comparisons test.

To evaluate the in vivo effect of NH_4_OH, MDA-MB-231 cells were introduced into the inguinal mammary fat pads of female athymic nude mice to establish xenograft mammary tumors. Once tumors reached the treatment threshold, mice received intratumoral injections of NH_4_OH or PBS control, and tumor growth was monitored by caliper measurement. At the end of the study, tumor and kidney tissues were collected for histological and molecular analyses, including immunohistochemistry, Western blotting, and qPCR (Figure 3A).

Total tumor burden was assessed over the experimental period in tumor-bearing mice treated with PBS (T-PBS) or NH_4_OH (T-NH). Both groups showed progressive tumor growth over time. Although intratumoral NH_4_OH treatment did not significantly reduce total tumor burden compared with PBS-treated controls, the T-NH group showed a trend toward lower tumor burden at later time points (Figure 3B and Figure S11). No treatment-related morbidity or mortality was observed during the study. Furthermore, longitudinal and endpoint body weight measurements did not differ significantly between the experimental groups (Supplementary Figure S6), indicating that intratumoral NH_4_OH treatment was well tolerated under the conditions examined.

**Figure 4:**
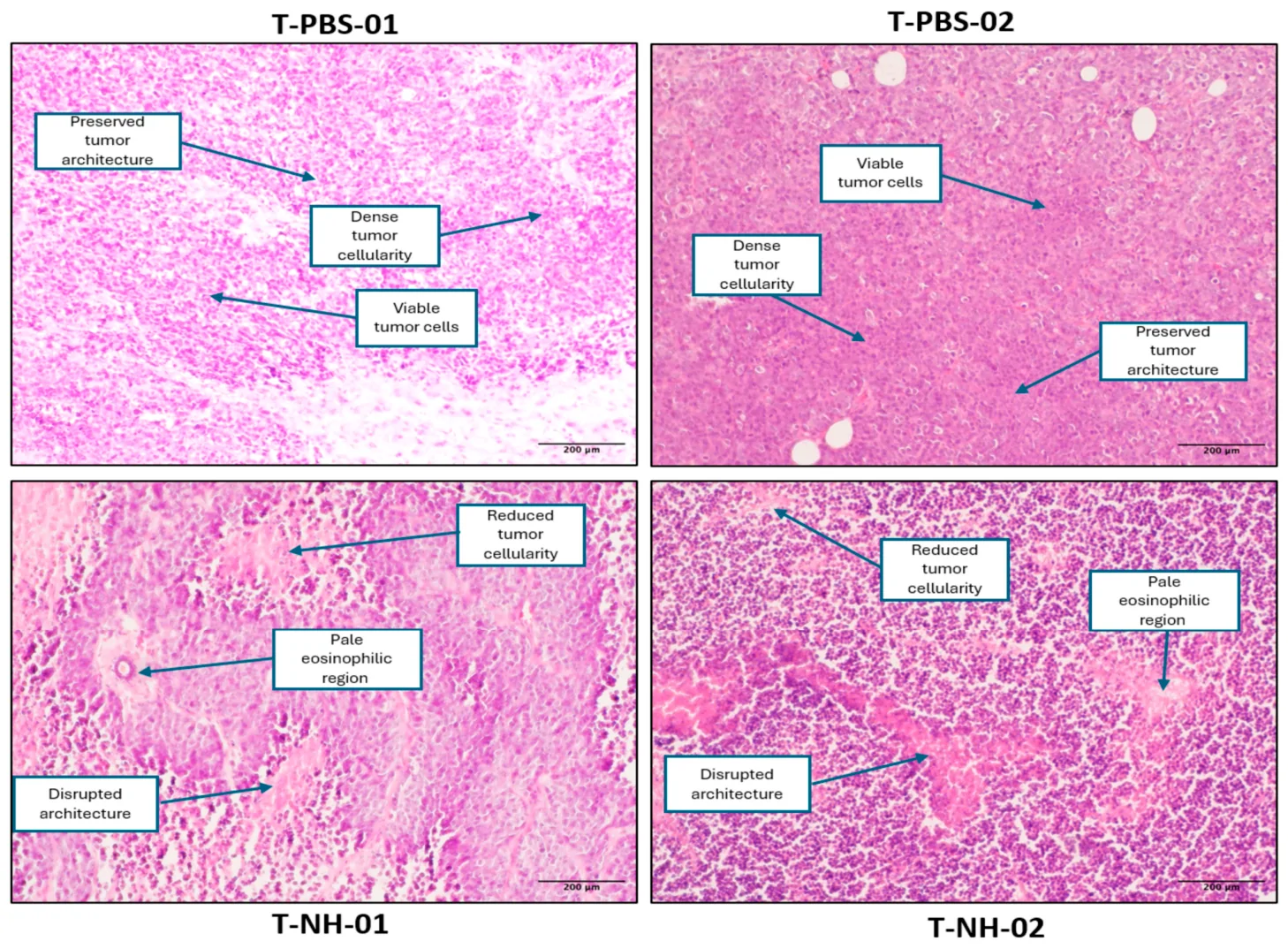
H&E staining of tumor tissues from vehicle control and NH_4_OH-treated tumor groups. Representative H&E-stained tumor sections from T-PBS and T-NH groups. The top panel shows PBS-treated tumors, and the bottom panel shows NH_4_OH-treated tumors. T-PBS tumors showed relatively dense tumor cellularity, whereas T-NH tumors showed altered tissue architecture with increased pale eosinophilic, cell-poor regions. Images were acquired using a 20× objective (200× total magnification). Scale bar = 200 µm.

To assess whether intratumoral NH_4_OH treatment was associated with histological changes in tumor tissue, H&E-stained sections from T-PBS and T-NH tumors were examined. T-PBS tumors showed relatively dense tumor cellularity with preserved tumor architecture. In contrast, T-NH tumors displayed altered tissue morphology, including increased pale eosinophilic regions and reduced cellular density, suggestive of treatment-associated tumor tissue disruption (Figure 4). Results are shown in Figure 4.

#### 3.2.2. Intratumoral NH_4_OH Attenuates Inflammatory Signaling in Xenograft Tumors

**Figure 5:**
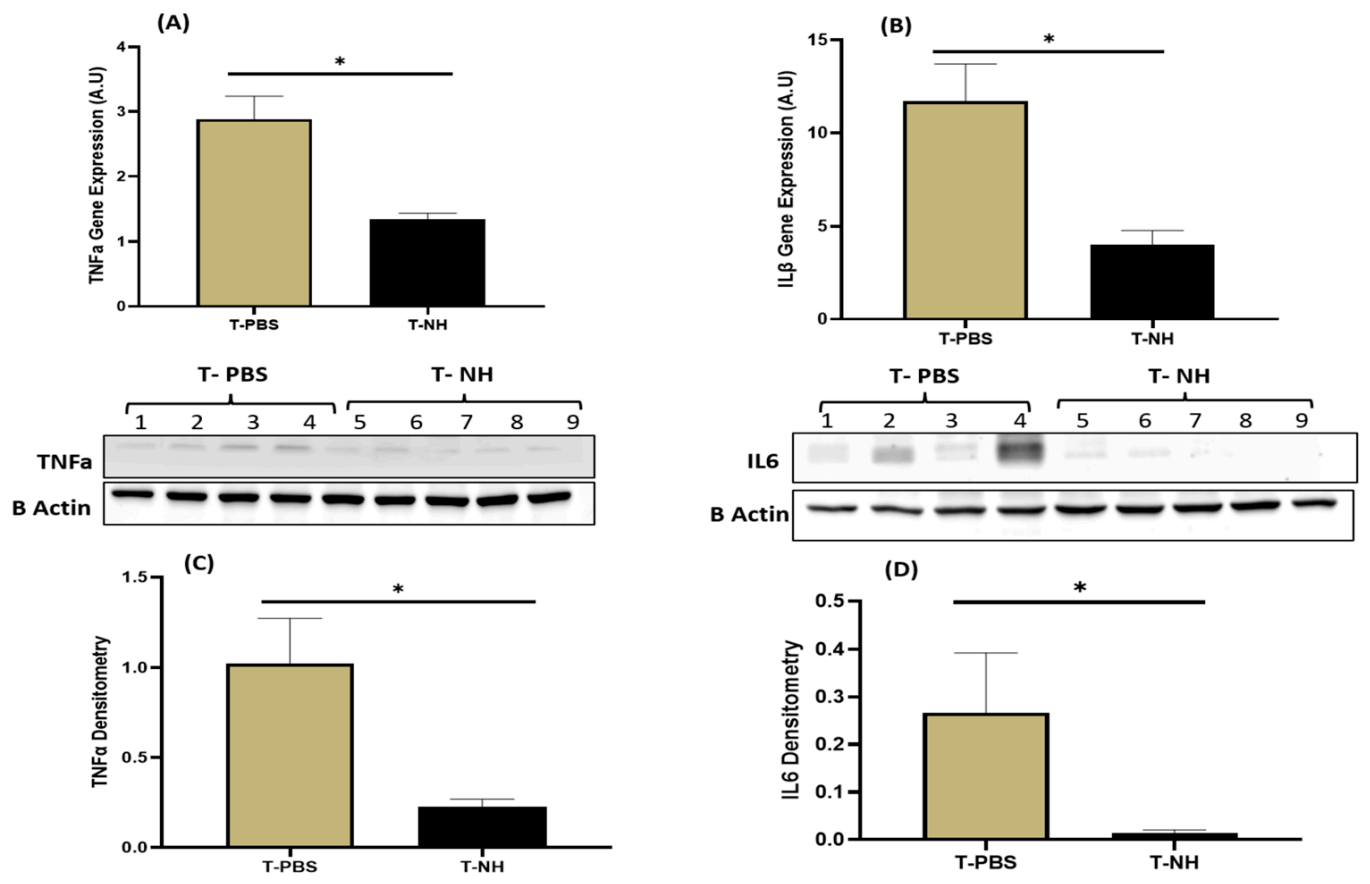
qPCR and Western blot analysis of TNFα, IL-1β and IL-6 expression in xenograft tumors. Tumor tissues from T-PBS and T-NH groups were analyzed for inflammatory marker expression. (**A**) *TNFα* and (**B**) *IL-1β* gene expression was assessed by qPCR. Representative Western blots are shown for TNFα and IL-6 with β-actin as the loading control. Densitometric quantification of (**C**) TNFα and (**D**) IL-6 protein expression was normalized to β-actin. Data are presented as mean ± SEM. Statistical significance was determined using an unpaired *t*-test. * *p* < 0.05 versus T-PBS.

To determine whether intratumoral NH_4_OH treatment modulates tumor-associated inflammatory signaling, expression of key inflammatory mediators was evaluated in tumor tissues from T-PBS and T-NH groups. qPCR analysis showed significantly reduced *TNFα* and *IL-1β* gene expression in NH_4_OH-treated tumors compared with PBS-treated controls (Figure 5A,B). Consistent with these transcriptional changes, Western blot analysis demonstrated significant decreased TNFα and IL-6 protein expression in the T-NH group, as confirmed by densitometric quantification normalized to β-actin (Figure 5C,D).

**Figure 6:**
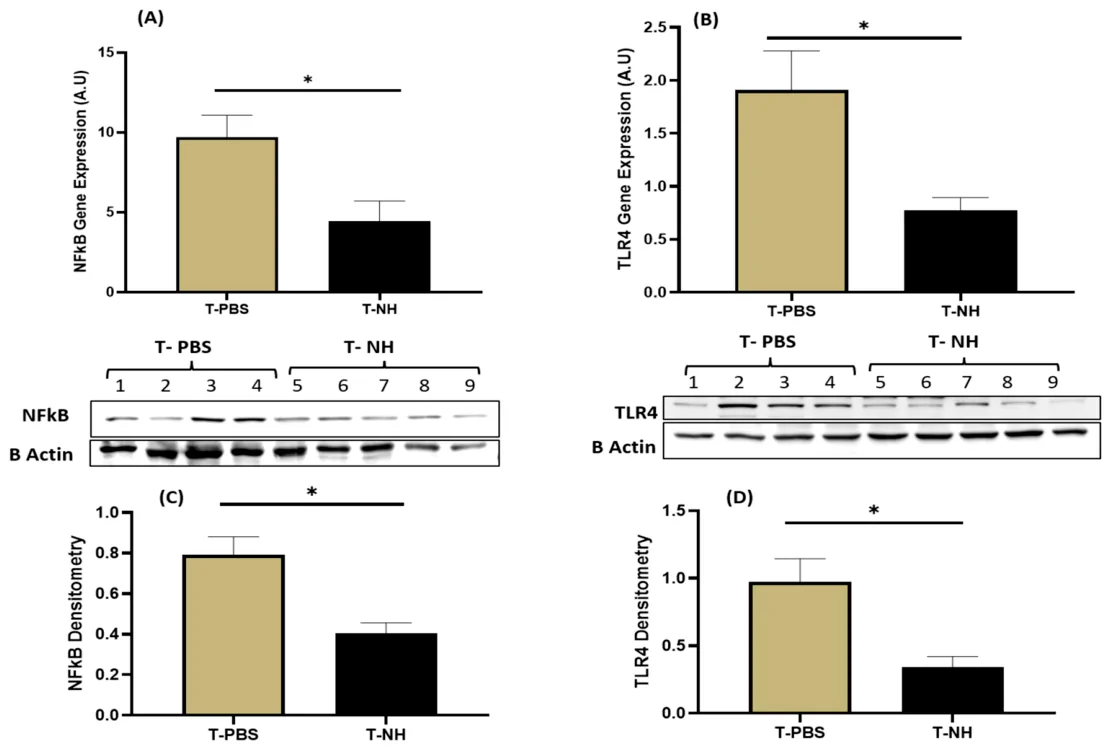
qPCR and Western blot analysis of NF-κB and TLR4 expression in xenograft mammary tumors. Tumor tissues from T-PBS and T-NH groups were analyzed for inflammatory signaling markers. (**A**) *NF-κB* and (**B**) *TLR4* gene expression were assessed by qPCR. Representative Western blots are shown for NF-κB and TLR4 with β-actin as the loading control. Densitometric quantification of (**C**) NF-κB and (**D**) TLR4 protein expression was normalized to β-actin. Data are presented as mean ± SEM. Statistical significance was determined using an unpaired *t*-test. * *p* < 0.05 versus T-PBS.

To further assess upstream inflammatory signaling, NF-κB and TLR4 expression were also examined. NH_4_OH-treated tumors showed significantly reduced *NF-κB* and *TLR4* gene expression compared with PBS-treated tumors (Figure 6A,B). Representative Western blots and densitometric analysis further demonstrated significantly decreased NF-κB and TLR4 protein expression in the T-NH group (Figure 6C,D). Together, these findings indicate that intratumoral NH_4_OH treatment attenuates inflammatory signaling in MDA-MB-231 xenograft tumors, involving both TLR4/NF-κB signaling and downstream pro-inflammatory cytokine expression.

#### 3.2.3. Intratumoral NH_4_OH Enhances Apoptotic Marker Expression in Xenograft Mammary Tumors

**Figure 7:**
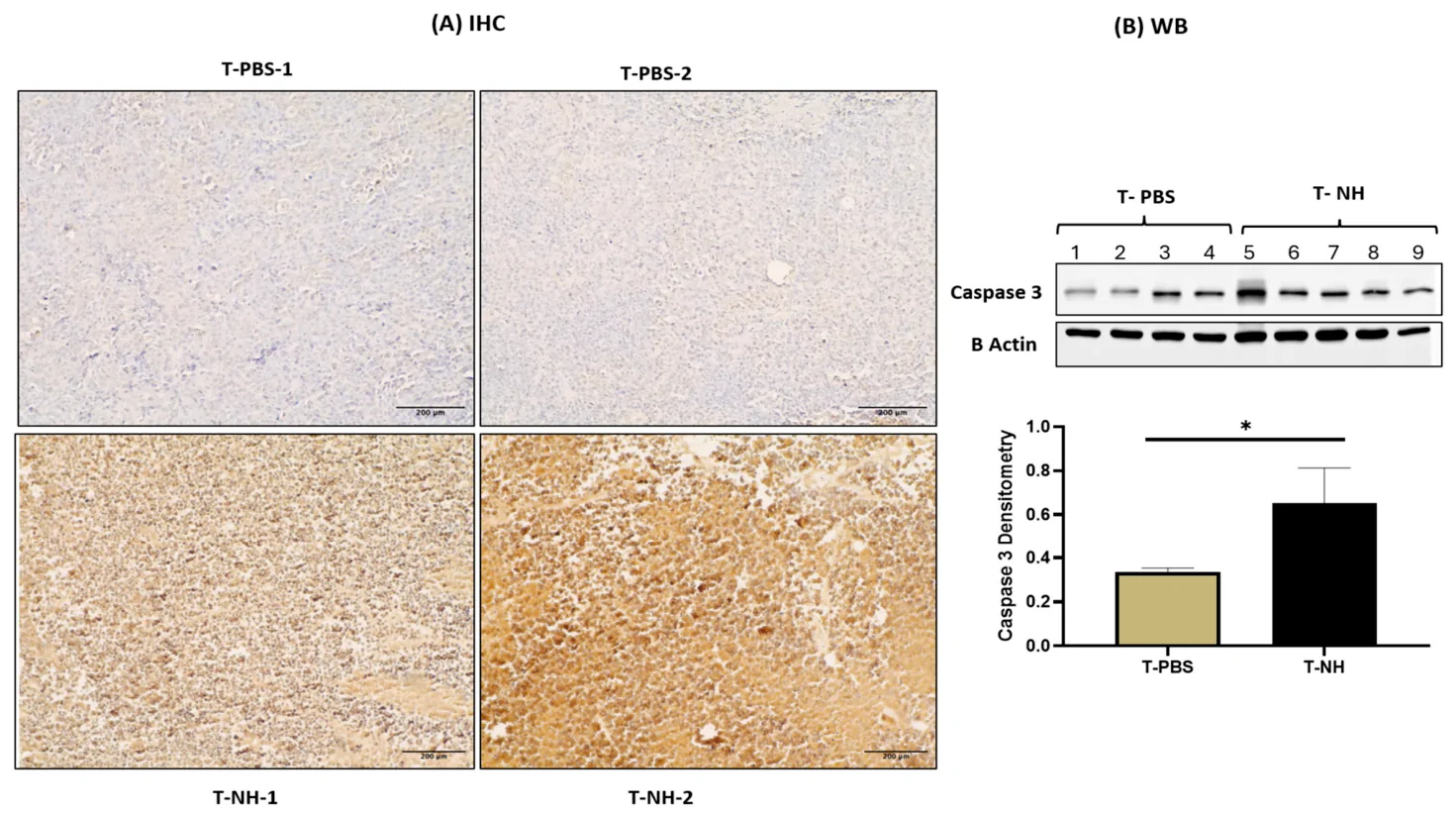
Caspase-3 expression by IHC and Western blot analysis in xenograft tumors. (**A**) Representative Caspase-3 IHC staining in tumor tissues from T-PBS and T-NH groups. The top panel shows T-PBS tumors, and the bottom panel shows T-NH tumors. Brown DAB staining indicates Caspase-3-positive immunoreactivity, with hematoxylin nuclear counterstain. (**B**) Representative Western blot and densitometric quantification of Caspase-3 protein expression normalized to β-actin. Data are presented as mean ± SEM. Statistical significance was determined using an unpaired *t*-test. * *p* < 0.05 versus T-PBS. Images were acquired using a 20× objective (200× total magnification). Scale bar = 200 µm.

To determine whether NH_4_OH treatment promotes apoptotic signaling in xenograft tumors, Caspase-3 expression was evaluated by immunohistochemistry (IHC) and Western blot analysis. Representative Caspase-3 IHC images showed weak staining in T-PBS tumors, whereas T-NH tumors displayed increased DAB (brown) staining, indicating greater Caspase-3 immunoreactivity following NH_4_OH treatment (Figure 7A). Consistent with the IHC findings, Western blot analysis showed significantly increased Caspase-3 protein expression in the T-NH group compared with the T-PBS group. Densitometric quantification confirmed increased Caspase-3 expression following NH_4_OH treatment (Figure 7B). These findings suggest that intratumoral NH_4_OH enhances apoptotic signaling in MDA-MB-231 xenograft tumors. Quantitative measure of percent positive immunoreactivity analyzed in IHC samples is found in Figure S2 of Supplementary Materials.

**Figure 8:**
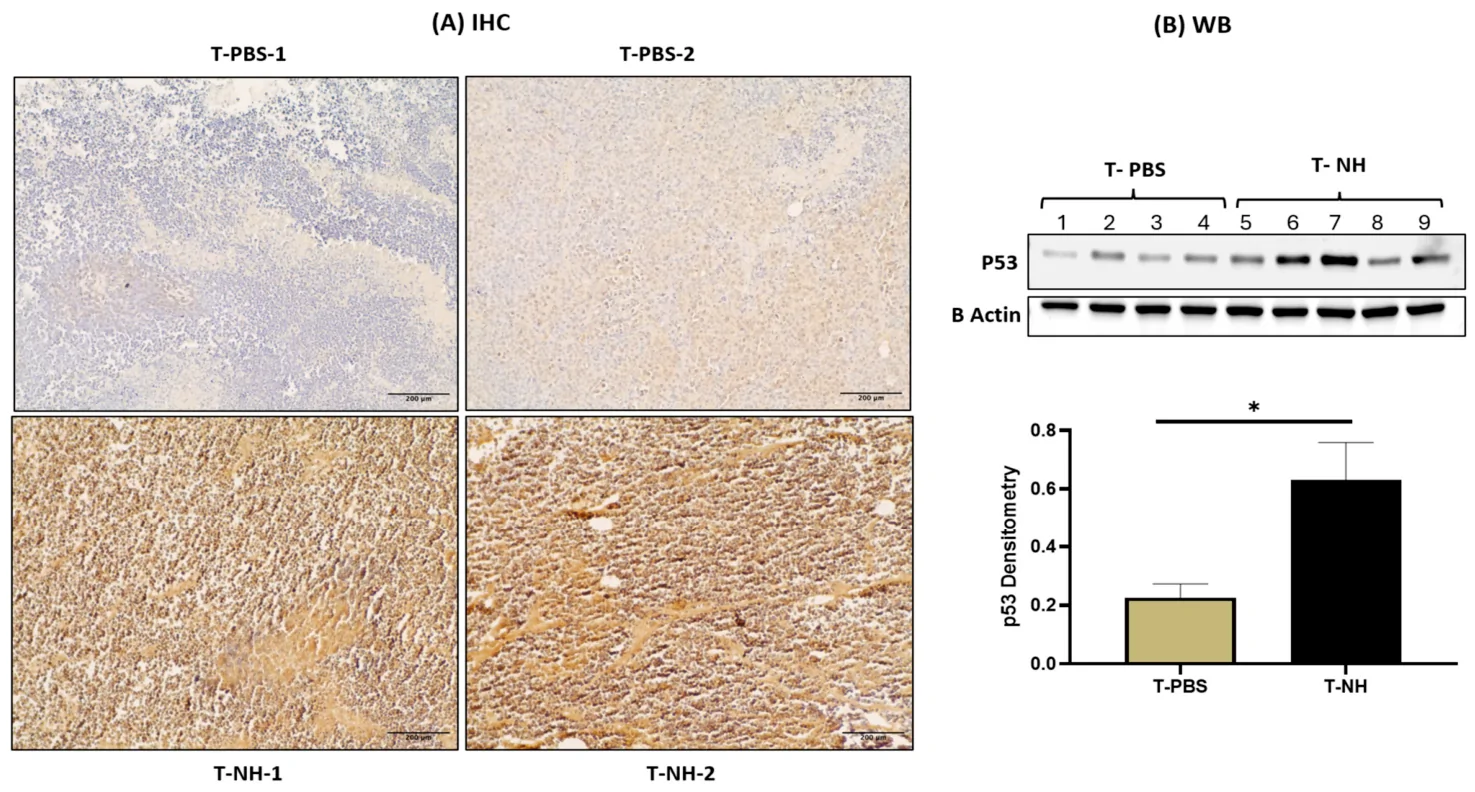
p53 immunohistochemistry and Western blot analysis in xenograft tumors. (**A**) Representative p53 IHC staining in tumor tissues from T-PBS and T-NH groups. The top panel shows T-PBS tumors, and the bottom panel shows T-NH tumors. Brown DAB staining indicates p53-positive immunoreactivity, with hematoxylin nuclear counterstain. (**B**) Representative Western blot and densitometric quantification of p53 protein expression normalized to β-actin. Data are presented as mean ± SEM. Statistical significance was determined using an unpaired *t*-test. * *p* < 0.05 versus T-PBS. Images were acquired using a 20× objective (200× total magnification). Scale bar = 200 µm.

To further evaluate apoptotic/stress-associated signaling in tumor tissues, p53 expression was examined by IHC and Western blot analysis. Representative p53 IHC images showed weaker staining in T-PBS tumors, whereas T-NH tumors displayed increased brown DAB staining, indicating greater p53 immunoreactivity following NH_4_OH treatment (Figure 8A). Consistent with the IHC findings, Western blot analysis showed increased p53 protein expression in the T-NH group compared with the T-PBS group. Densitometric quantification confirmed significantly increased p53 expression following NH_4_OH treatment (Figure 8B). Quantitative measure of percent positive immunoreactivity analyzed in IHC samples is found in Figure S3 of Supplementary Materials. Because MDA-MB-231 cells harbor a mutant TP53 genotype, the increased p53 immunoreactivity could be due to altered mutant p53 expression associated with cellular stress rather than restoration of canonical wild-type p53 tumor suppressor activity. Nevertheless, when considered together with the increased caspase-3 expression, these findings are consistent with enhanced apoptotic and cellular stress-associated signaling following NH_4_OH treatment.

**Figure 9:**
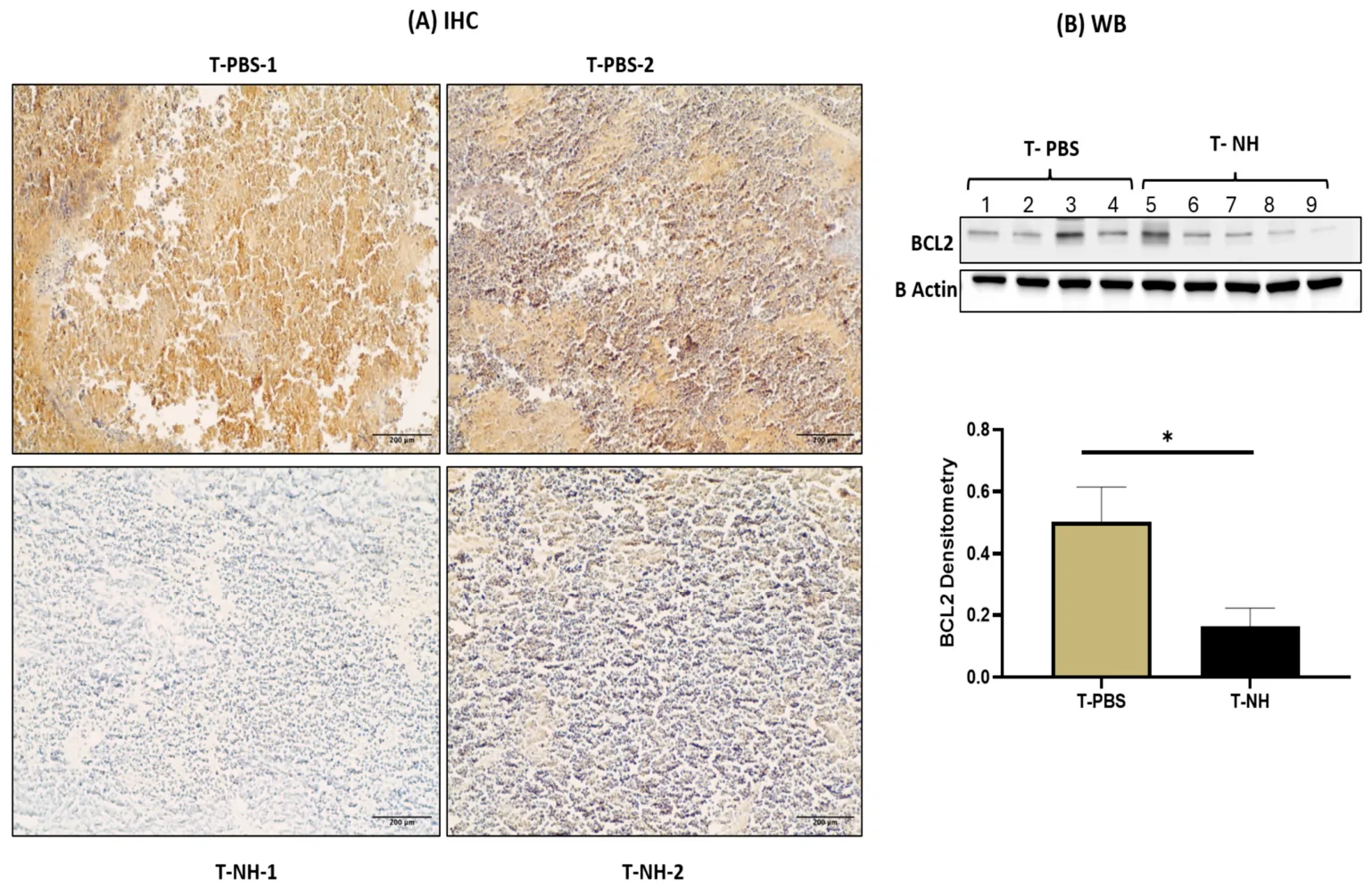
BCL2 IHC and Western blot analysis in xenograft tumors. (**A**) Representative IHC staining for BCL2 in tumor tissues from T-PBS and T-NH groups. The top panel shows T-PBS tumors, and the bottom panel shows T-NH tumors. Brown DAB staining indicates BCL2-positive immunoreactivity, with hematoxylin nuclear counterstain. (**B**) Representative Western blot and densitometric quantification of BCL2 protein expression normalized to β-actin. Data are presented as mean ± SEM. Statistical significance was determined using an unpaired *t*-test. * *p* < 0.05 versus T-PBS. Images were acquired using a 20× objective (200× total magnification). Scale bar = 200 µm.

To determine whether NH_4_OH treatment induced apoptosis in tumors, BCL2 expression was evaluated by IHC and Western blot analysis. Representative IHC images showed stronger BCL2 staining in T-PBS tumors whereas, T-NH tumors displayed reduced BCL2 immunoreactivity following NH_4_OH treatment (Figure 9A). Consistent with the IHC findings, Western blot analysis showed decreased BCL2 protein expression in the T-NH group compared with the T-PBS group. Densitometric quantification confirmed significantly reduced BCL2 expression following NH_4_OH treatment (Figure 9B) in the T-NH group. Quantitative measure of percent positive immunoreactivity analyzed in IHC samples is found in Figure S4 of Supplementary Materials. Together with increased Caspase-3 and p53 expression, these findings suggest that NH_4_OH treatment induces a pro-apoptotic shift in MDA-MB-231 xenograft tumors.

#### 3.2.4. NH_4_OH Treatment Reduces Cell Proliferation in Xenograft Tumors

**Figure 10:**
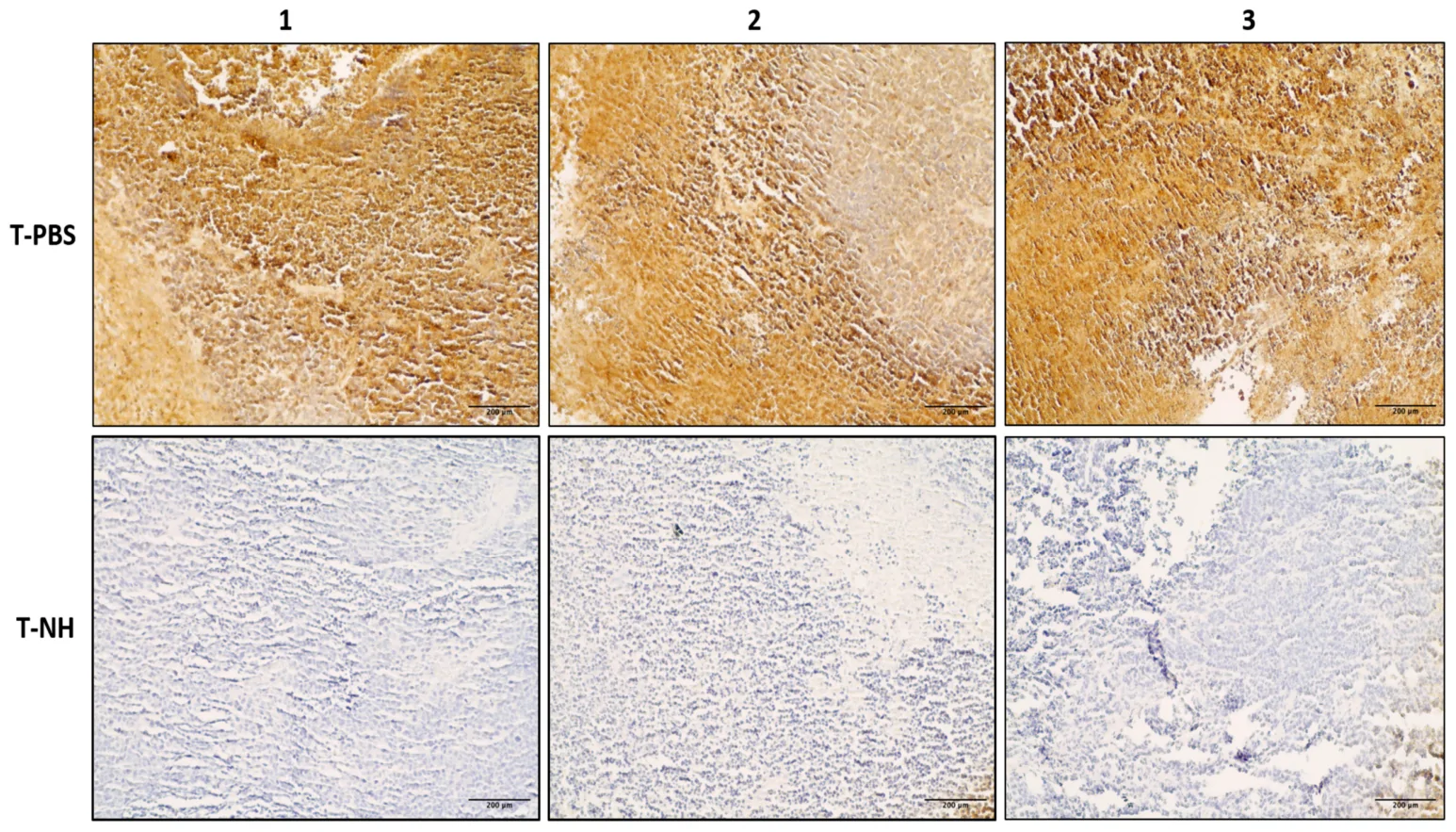
Ki-67 analysis by IHC in tumor tissues from T-PBS and T-NH groups. Representative Ki-67 IHC staining in xenograft tumor sections from T-PBS and T-NH groups. The top panel shows PBS-treated tumors, and the bottom panel shows NH_4_OH-treated tumors. Brown DAB staining indicates Ki-67-positive proliferating cells, with hematoxylin nuclear counterstain. T-PBS tumors showed stronger Ki-67 immunoreactivity, whereas T-NH tumors showed reduced Ki-67 staining. Images were acquired using a 20× objective (200× total magnification). Scale bar = 200 µm.

To assess whether intratumoral NH_4_OH treatment affected tumor cell proliferation, Ki-67 expression was evaluated by IHC in tumor tissues from T-PBS and T-NH groups. Representative Ki-67-stained sections showed intense immunoreactivity in T-PBS tumors, whereas T-NH tumors showed virtually no Ki-67 staining (Figure 10). Quantitative measure of percent positive immunoreactivity analyzed in IHC samples is found in Figure S5 of Supplementary Materials. These findings indicate that NH_4_OH treatment decreases proliferative activity in MDA-MB-231 xenograft tumors.

### 3.3. Effects of Intratumoral Treatment with NH_4_OH on Renal Function

#### 3.3.1. Intratumoral NH_4_OH Attenuates Renal Inflammatory Marker Expression Without Activating NF-κB Signaling

**Figure 11:**
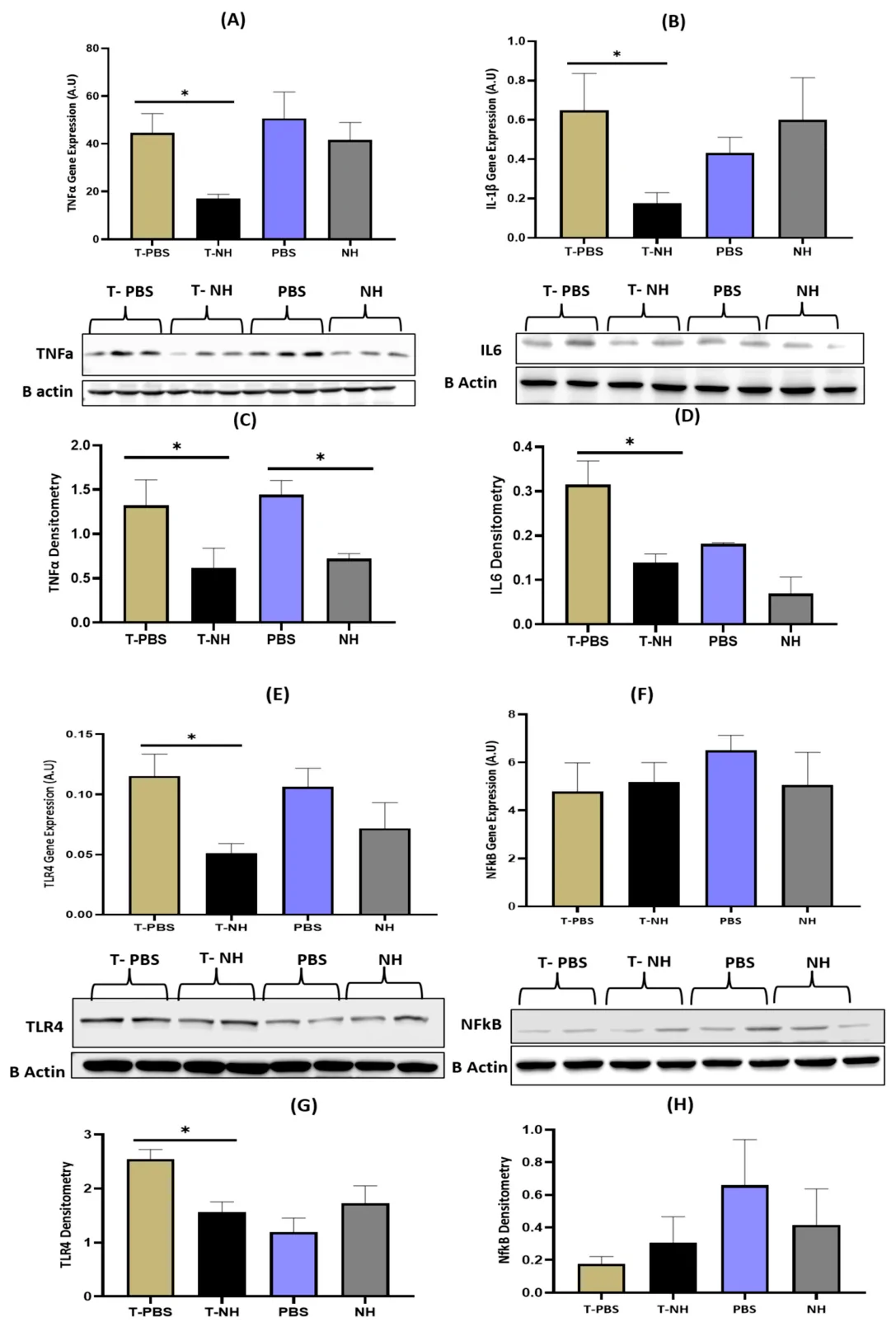
Systemic effects of intratumoral NH_4_OH on renal inflammatory marker expression. Kidney tissues from T-PBS and T-NH tumor groups, and PBS and NH control groups were analyzed to evaluate the systemic effects of intratumoral NH_4_OH treatment. (**A**) *TNFα* and (**B**) *IL-1β* gene expression were determined by qPCR. Representative Western blots and densitometric quantification of (**C**) TNFα and (**D**) IL-6 protein expression normalized to β-actin are shown. (**E**) *TLR4* and (**F**) *NF-κB* gene expression were assessed by qPCR. Representative Western blots and densitometric quantification of (**G**) TLR4 and (**H**) NF-κB protein expression normalized to β-actin are shown. TNFα, IL-6, and TLR4 expression were reduced in kidneys from NH_4_OH-treated tumor-bearing mice compared with PBS-treated tumor-bearing mice, whereas NF-κB expression was not significantly different among the experimental groups. Data are presented as mean ± SEM. Statistical significance was determined using one-way ANOVA followed by Fisher’s LSD post hoc test. * *p* < 0.05 versus T-PBS.

To evaluate the potential systemic effects of intratumoral NH_4_OH treatment, kidney tissues were analyzed for inflammatory cytokines and signaling molecules associated with renal inflammation. Gene expression of *TNFα*, *NF-κB, TLR4* and *IL1β*, and protein expression of TNFα, NF-κB, TLR4, and IL-6, were examined in T-PBS and T-NH tumor groups, and PBS and NH control groups. qPCR analysis demonstrated that renal *TNFα* and *IL-1β* gene expression were significantly reduced in the T-NH group compared with the T-PBS group (Figure 11A,B). Consistent with these findings, Western blot analysis showed significantly reduced TNFα and IL-6 protein expression in kidneys from T-NH mice compared with T-PBS mice (Figure 11C,D).

To assess status of upstream inflammatory signaling, TLR4 and NF-κB expression were evaluated. Both qPCR and Western blot analyses demonstrated significantly reduced TLR4 expression in the T-NH group compared with the T-PBS group (Figure 11E,G). In contrast, NF-κB gene and protein expression did not differ significantly between the experimental groups (Figure 11F,H).

#### 3.3.2. Effects of Intratumoral NH_4_OH Treatment on Expression of Apoptotic Proteins in Kidney

##### Intratumoral NH_4_OH Treatment Reduces Renal Caspase-3 Expression

**Figure 12:**
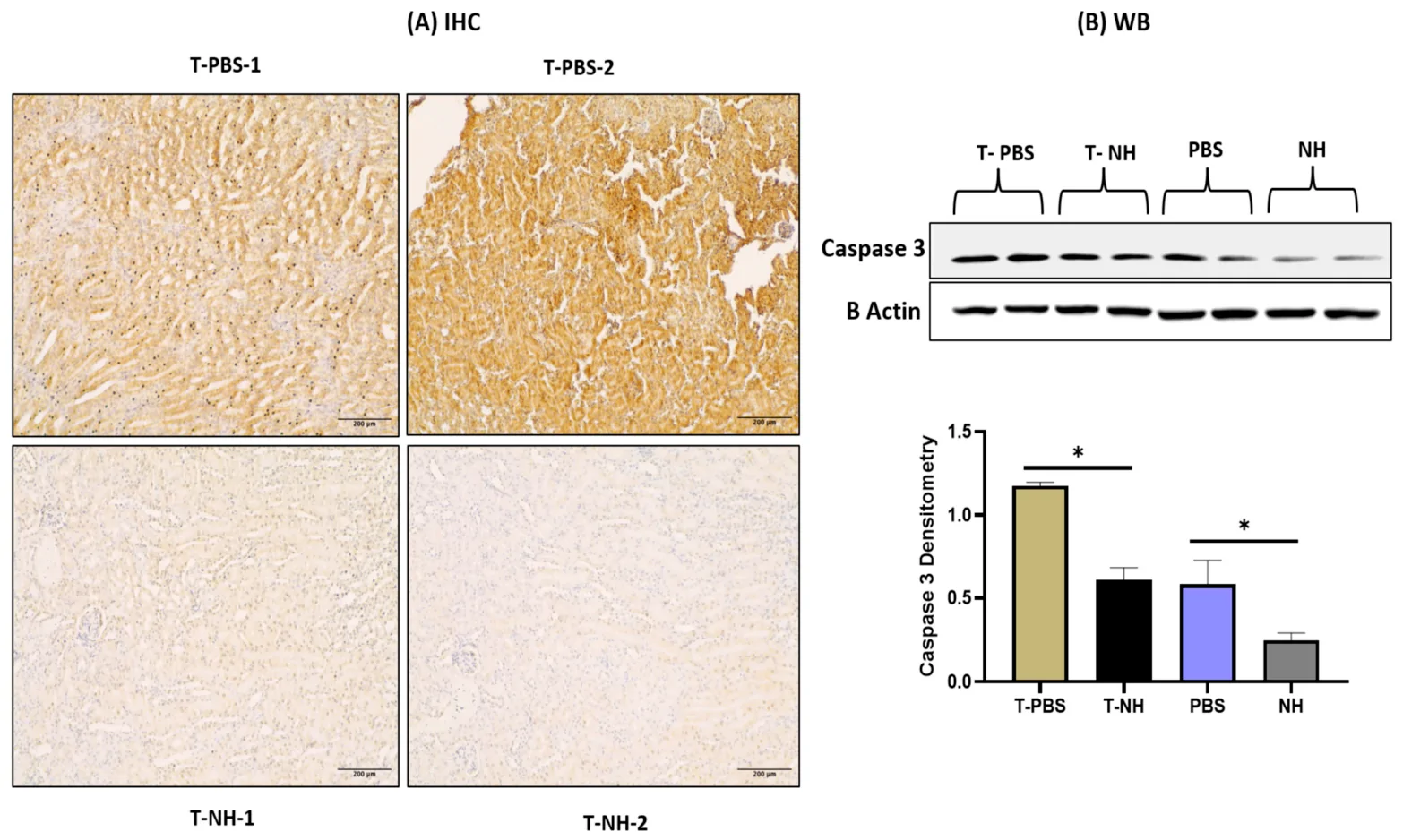
Renal Caspase-3 expression following intratumoral NH_4_OH treatment. (**A**) Representative Caspase-3 IHC staining in kidney tissues from T-PBS and T-NH groups. The top panel shows T-PBS kidney tissue, and the bottom panel shows T-NH kidney tissue. Strong DAB staining indicates Caspase-3-positive immunoreactivity. (**B**) Representative Western blot and densitometric quantification of renal Caspase-3 protein expression normalized to β-actin. Caspase-3 expression was significantly reduced in the T-NH group compared with the T-PBS group. Data are presented as mean ± SEM. Statistical significance was determined using one-way ANOVA followed by Fisher’s LSD post hoc test. * *p* < 0.05 versus T-PBS. Images were acquired using a 20× objective (200× total magnification). Scale bar = 200 µm.

To determine the potential systemic effects of intratumoral NH_4_OH treatment on kidney tissue, Caspase-3 expression was examined by IHC and Western blot analysis. Representative kidney sections from T-PBS mice showed strong Caspase-3 immunoreactivity in contrast to kidneys from T-NH mice (Figure 12A). Consistent with the IHC findings, Western blot analysis showed decreased renal Caspase-3 protein expression in the T-NH group compared with the T-PBS group. Densitometric quantification confirmed a significant reduction in Caspase-3 expression following NH_4_OH treatment (Figure 12B). These findings suggest that intratumoral NH_4_OH treatment does not promote renal apoptotic signaling and may reduce tumor-associated renal apoptotic stress.

##### Intratumoral NH_4_OH Does Not Significantly Alter Renal p53 Protein Expression

**Figure 13:**
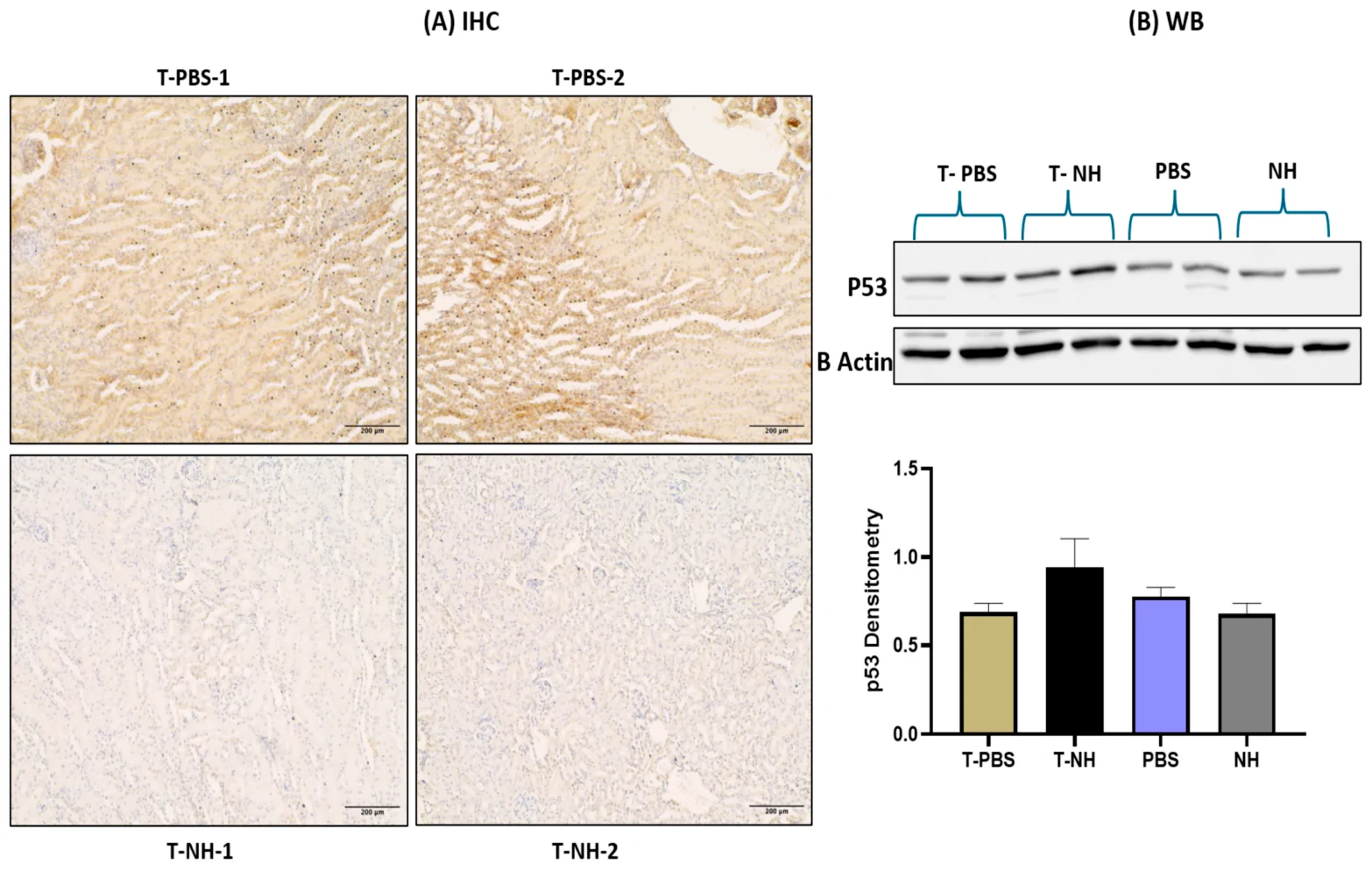
Analysis of renal p53 expression following intratumoral NH_4_OH treatment. (**A**) Representative p53 IHC staining in kidney tissues from T-PBS and T-NH groups. The top panel shows T-PBS kidneys, and the bottom panel shows T-NH kidneys. Brown DAB staining indicates p53-positive immunoreactivity. Hematoxylin is used as nuclear counterstain. (**B**) Representative Western blot and densitometric quantification of renal p53 protein expression normalized to β-actin. No significant difference in p53 protein expression was observed among groups. Data are presented as mean ± SEM. Statistical significance was determined using one-way ANOVA followed by Fisher’s LSD post hoc test. Images were acquired using a 20× objective (200× total magnification), Scale bar = 200 µm.

To investigate the potential systemic effects of intratumoral NH_4_OH treatment on renal p53 expression, p53 was evaluated in kidney tissues by IHC and Western blot analysis. Representative p53 IHC images showed strong staining in T-PBS kidneys, while T-NH kidneys showed low p53 immunoreactivity (Figure 13A). Interestingly however, Western blot analysis and densitometric quantification showed no significant difference in renal p53 protein expression among the experimental groups (Figure 13B).

##### Intratumoral NH_4_OH Increases Renal BCL2 Expression

**Figure 14:**
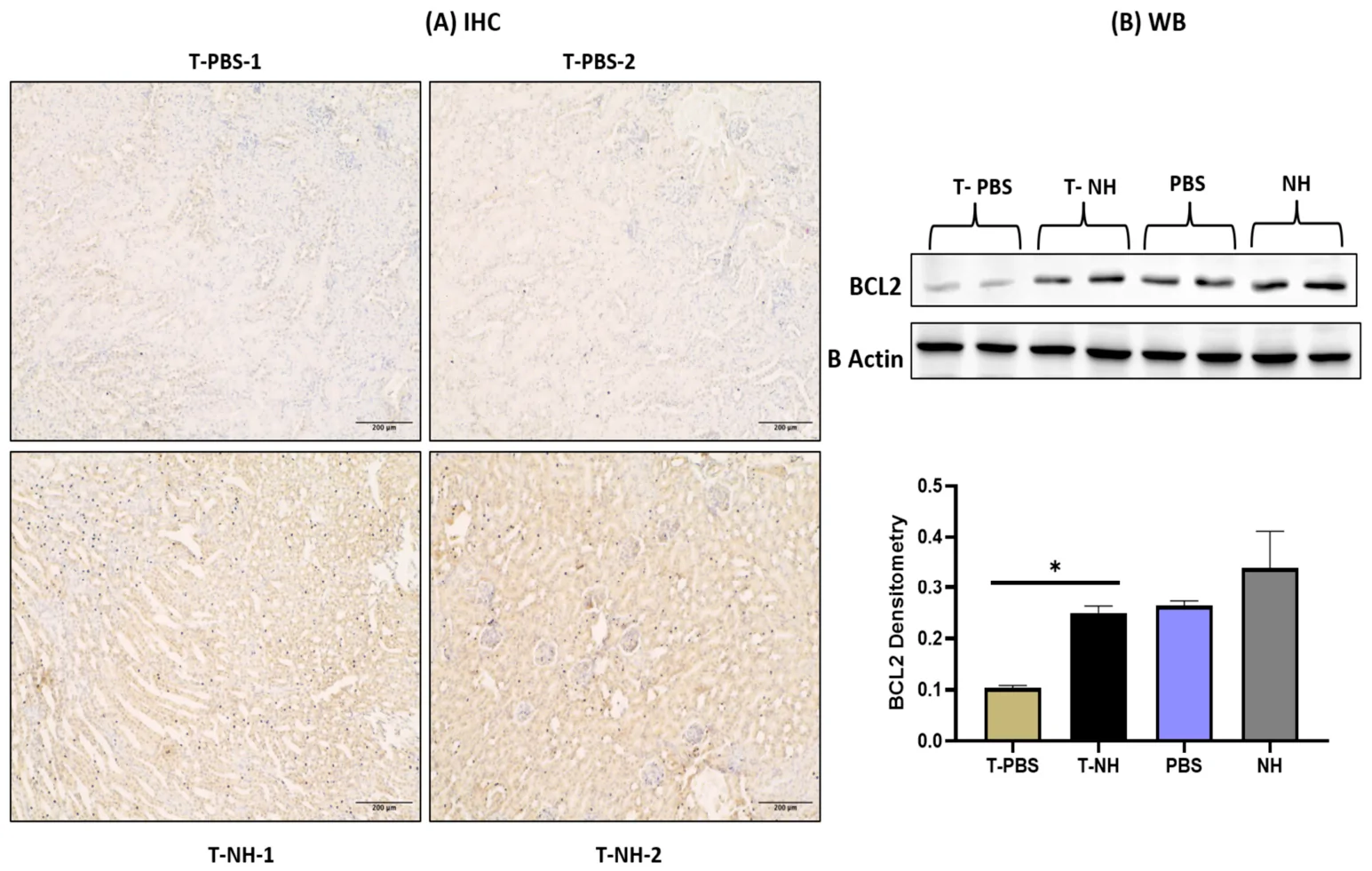
Analysis of renal BCL2 protein expression following intratumoral NH_4_OH treatment. (**A**) Representative BCL2 IHC staining in kidney tissues from T-PBS and T-NH groups. The top panel shows T-PBS kidneys, and the bottom panel shows T-NH kidneys. Brown DAB staining indicates BCL2-positive immunoreactivity, with hematoxylin as nuclear counterstain. (**B**) Representative Western blot and densitometric quantification of renal BCL2 protein expression normalized to β-actin. BCL2 expression was significantly increased in the T-NH group compared with T-PBS. Data are presented as mean ± SEM. Statistical significance was determined using one-way ANOVA followed by Fisher’s LSD post hoc test. * *p* < 0.05 versus T-PBS. Images were acquired using a 20× objective (200× total magnification). Scale bar = 200 µm.

Intratumoral NH_4_OH treatment on renal apoptotic regulation was further analyzed by determining BCL2 expression by IHC and Western blot analysis. Interestingly, representative BCL2 IHC images showed stronger BCL2 immunoreactivity in T-NH kidneys compared with T-PBS kidneys (Figure 14A). Consistent with the IHC findings, Western blot analysis and densitometric quantification demonstrated a significant increase in renal BCL2 protein expression in the T-NH group compared with the T-PBS group (Figure 14B). Together with the observed reduction in renal Caspase-3 expression, these findings suggest that intratumoral NH_4_OH treatment does not induce renal apoptotic signaling and may support preservation of renal cell survival signaling.

#### 3.3.3. Systemic Effects of Intratumoral NH_4_OH Include Enhanced Renal Ammonia Transporter Expression

**Figure 15:**
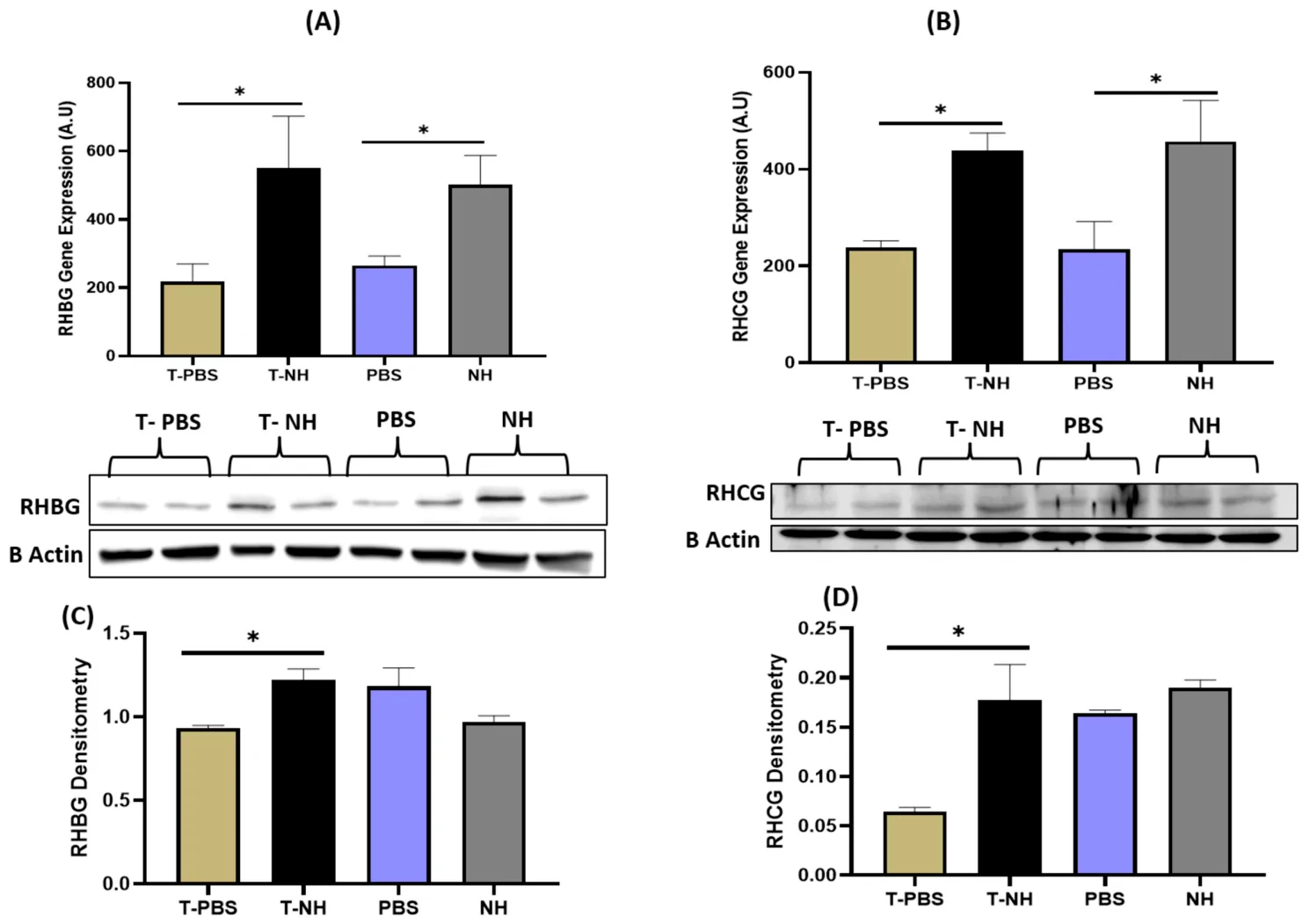
Analysis of renal RHBG and RHCG expression following intratumoral NH_4_OH treatment. Kidney tissues from T-PBS, T-NH, PBS control, and NH control groups were analyzed for ammonia transporter expression. (**A**) *RHBG* and (**B**) *RHCG* gene expression were assessed by qPCR. Representative Western blots are shown for RHBG and RHCG with β-actin as the loading control. Densitometric quantification of (**C**) RHBG and (**D**) RHCG protein expression was normalized to β-actin. Data are presented as mean ± SEM. Statistical significance was determined using one-way ANOVA followed by Fisher’s LSD post hoc test. * *p* < 0.05 versus T-PBS.

Due to the importance of ammonium ions in metabolism, coupled with the dysfunction metabolism of tumor cells, it was important to determine the effects of intratumoral NH_4_OH treatment on renal ammonia-handling pathways. Thus, expression of the ammonia transporters RHBG and RHCG were examined in kidney tissues. qPCR analysis showed increased *RHBG* and *RHCG* gene expression in the T-NH group compared with the T-PBS group (Figure 15A,B). Western blot analysis further demonstrated increased RHBG and RHCG protein expression in kidney tissues, with densitometric quantification showing higher transporter expression in the T-NH group relative to the T-PBS group (Figure 15C,D). These findings suggest that intratumoral NH_4_OH treatment may modulate renal ammonia transporter expression as part of the systemic response to treatment.

#### 3.3.4. Intratumoral NH_4_OH Preserves Renal Morphology While Attenuating Distal Tubular Alterations

**Figure 16:**
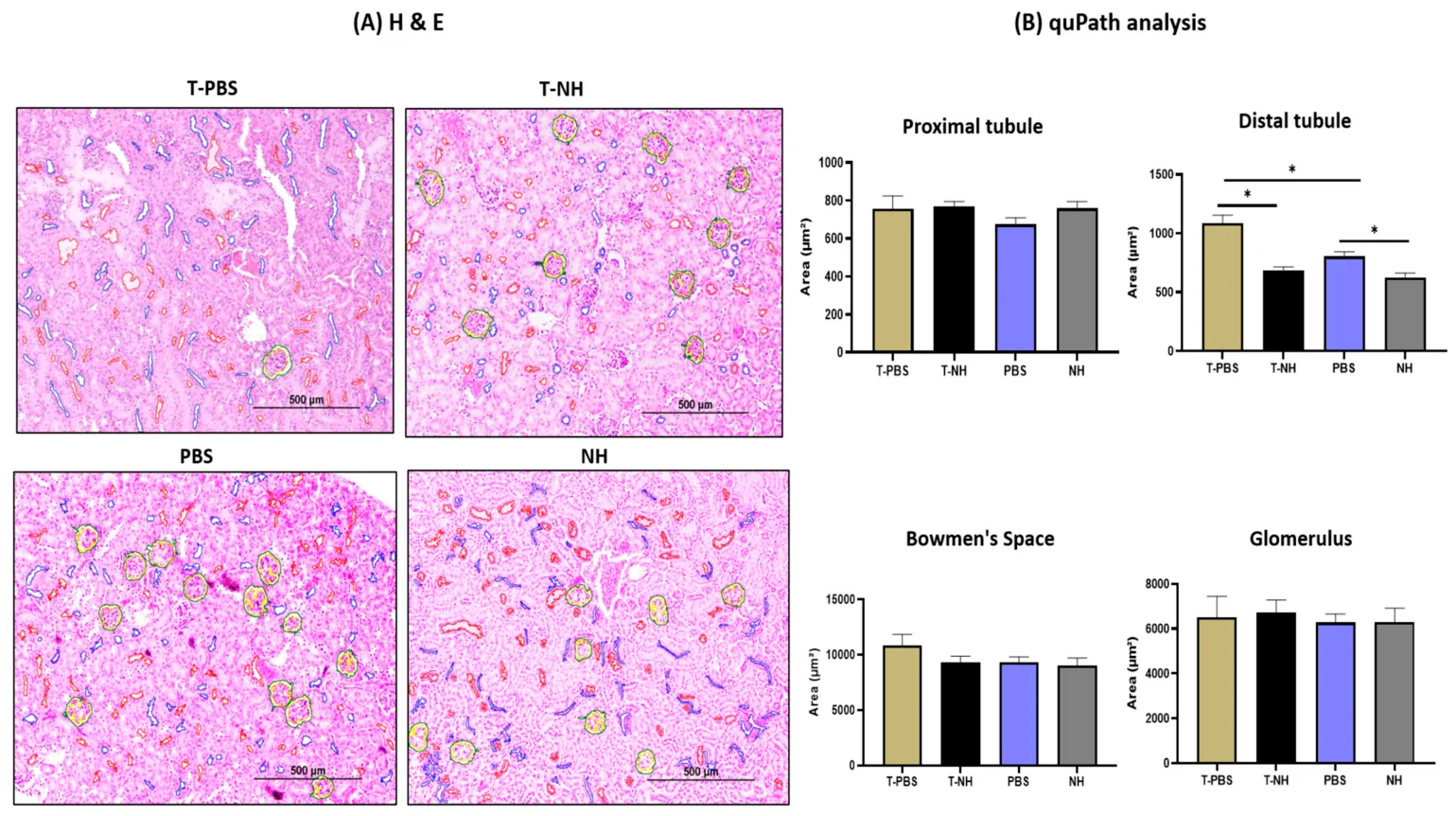
Kidney H&E staining and morphometric analysis following intratumoral NH_4_OH treatment. (**A**) Representative H&E-stained kidney sections from T-PBS, T-NH, PBS control, and NH control groups. 10X H&E-stained kidney sections with QuPath polygon annotations denoting proximal tubules (red), distal tubules (blue), glomeruli (yellow), and Bowman’s space (green). Annotations were used to quantify the area of these four components across treatment groups on a 500 µm scale bar. The top panel shows T-PBS and T-NH kidneys, and the bottom panel shows PBS control and NH control kidneys. (**B**) QuPath-based morphometric analysis of proximal tubules, distal tubules, Bowman’s space, and glomerular area. Distal tubule area was significantly reduced in both T-NH and NH groups compared with T-PBS and PBS control groups, respectively. Proximal tubules, Bowman’s space, and glomerular area showed no significant differences between groups. Data are presented as mean ± SEM. Statistical significance was determined using one-way ANOVA followed by Fisher’s LSD post hoc test. * *p* < 0.05.

Given our prior data from this study and our previous work [24,25,26,27,28,29] that NH_4_OH supplemented protein diets beneficially reduces cytokines and adipocytokines, it was important to analyze the morphological characteristics of the renal nephrons in greater detail. Thus, the Bowman’s space, glomerular area, proximal and distal convoluted tubule diameters were measured using a QuPath-based workflow (see Figure S1 for example output). Representative H&E-stained kidney sections revealed generally preserved renal histology across all experimental groups, with no evidence of overt tubular necrosis, glomerular injury, or inflammatory cell infiltration (Figure 16A). To objectively evaluate subtle structural changes, morphometric analysis was performed on the H&E sections using QuPath. While the quantitative analysis of the proximal tubular area, Bowman’s space, and glomerular area did not differ substantially among the experimental groups, there was a significant reduction in distal tubular area in the T-NH group compared with the T-PBS group, PBS control group compared with T-PBS group and in the NH control group compared with the PBS control group (Figure 16B). These findings indicate that while intratumoral NH_4_OH treatment does not produce overt renal histopathological injury, it does affect distal tubular diameter.

## 4. Discussion

The present study provides, to our knowledge, the first evaluation of ammonium hydroxide (NH_4_OH) as a localized intratumoral therapeutic strategy for breast cancer. By integrating complementary in vitro and in vivo approaches, we demonstrate that NH_4_OH affects key cancer-associated signaling pathways and reduces metabolic activity while showing no evidence of overt renal toxicity. Although intratumoral NH_4_OH treatment did not show significant reduction in tumor burden, substantial molecular and histological alterations were consistently observed within the NH-treated tumors. These changes, including reduced proliferative activity, enhanced apoptotic signaling, and decreased inflammatory marker expression, suggest a shift toward a less aggressive tumor phenotype, which may have implications in future studies for tumor responsiveness to standard therapies. These included increased Caspase-3 and p53 expression, reduced BCL2 and Ki-67 expression, and suppression of key inflammatory mediators, indicating that NH_4_OH altered the biological behavior of the tumor before measurable changes in tumor volume became evident. Therefore, the coordinated changes observed in the present pilot study support the idea that localized NH_4_OH exerts biologically meaningful antitumor effects despite the limited treatment duration and relatively small experimental cohort.

The in vitro studies provided a framework from which we could explore important physiologically relevant questions. We observed that NH_4_OH exposure inhibited the growth and metabolic activity of MDA-MB-231 breast cancer cells. In contrast, comparatively mild effects were observed on the non-tumorigenic MCF10A mammary epithelial cell line, suggesting that transformed cells may be intrinsically more susceptible to NH_4_OH-induced changes than normal epithelial cells. This differential sensitivity is biologically plausible because malignant cells undergo extensive metabolic reprogramming to sustain rapid proliferation, increased biosynthetic demands, and adaptation to the hostile tumor microenvironment [15,17,18,19]. In particular, triple-negative breast cancer cells exhibit a high dependence on glutamine metabolism, which provides carbon and nitrogen for nucleotide synthesis, redox homeostasis, and energy production [22,37,38,39]. Ammonia, generated during glutamine catabolism, has historically been regarded as a metabolic waste product. However, accumulating evidence indicates that it also functions as an important signaling metabolite capable of regulating autophagy, mitochondrial function, lysosomal homeostasis, and cellular adaptation within the tumor microenvironment [21,40,41,42]. While physiological concentrations of ammonia may support metabolic flexibility, excessive ammonia exposure has been reported to impair cellular homeostasis and activate stress-response pathways leading to growth arrest and apoptosis [43,44,45,46]. But these observations should be taken in the context of the study models because when extrapolating to humans, (1) excess ammonia exposure is very rare due to renal function and the ability to excrete excess ammonia wastes [47,48]; (2) the current obesity epidemic and poor dietary choices strongly infer low levels of metabolic acidosis in patient populations [49,50,51,52,53]. Thus, this is not likely physiological outcome. Consistent with these observations, NH_4_OH treatment significantly increased Annexin V-positive apoptotic cells after 12 and 24 h of exposure, whereas no significant effect was detected at 3 h (Supplementary Figure S9), indicating that NH_4_OH induces a progressive biological response rather than immediate nonspecific cytotoxic response. Although the present study did not directly evaluate intracellular pH, ammonia flux, or glutamine metabolism, the combined in vitro and in vivo findings are consistent with the possibility that localized NH_4_OH exposure may exceed the adaptive metabolic capacity of breast cancer cells, thereby contributing to a shift from cellular survival toward apoptosis. However, further mechanistic studies are required to confirm this hypothesis [21,22].

Intratumoral NH_4_OH administration produced localized molecular and histological alterations within the tumor microenvironment, characterized by reduced inflammatory signaling, decreased proliferation, enhanced apoptotic signaling, and disruption of tumor architecture. The pro-apoptotic effects of NH_4_OH were accompanied by coordinated suppression of inflammatory and proliferative signaling within the tumor. Chronic inflammation is widely recognized as a hallmark of breast cancer progression [54], where activation of the TLR4/NF-κB signaling axis stimulates production of pro-inflammatory cytokines such as TNFα, IL-1β and IL-6, thereby promoting tumor cell proliferation, angiogenesis, invasion, immune evasion, and resistance to therapy [55,56,57]. In the present study, gene expression of *TNFα*, *IL-1β* and protein expression of TNFα and IL-6 were significantly reduced following NH_4_OH treatment, together with decreased TLR4 expression, while NF-κB protein expression showed a modest downward trend. These molecular alterations were accompanied by increased Caspase-3 expression, reduced BCL2 expression, and markedly decreased Ki-67 immunoreactivity in tumor tissues, collectively suggesting that NH_4_OH suppresses signaling pathways that support tumor cell survival while simultaneously limiting proliferative activity. In the xenograft tumors, NH_4_OH treatment increased Caspase-3 expression and reduced BCL2 expression, supporting activation of apoptotic signaling. Tumors were established using MDA-MB-231 cells, which harbor a mutant TP53 genotype. Therefore, the observed increase in tumor p53 expression should be interpreted as altered mutant p53 expression associated with cellular stress rather than restoration of canonical wild-type p53 tumor suppressor activity [58,59,60,61]. Nevertheless, the coordinated increase in Caspase-3 together with reduced BCL2, attenuation of inflammatory signaling, and decreased Ki-67 expression supports a shift toward a less proliferative and more pro-apoptotic tumor phenotype following intratumoral NH_4_OH treatment. Collectively, these findings suggest that the antitumor effects of NH_4_OH extend beyond direct growth inhibition and involve simultaneous modulation of metabolic, inflammatory, and apoptotic pathways that are critical for breast cancer progression, aggressiveness and sensitivity to therapies [54,62,63,64].

These findings extend previous work from our laboratory investigating ammonium hydroxide enhancement (AHE) as a dietary intervention [26]. Our prior studies showed that AHE exerted beneficial systemic effects at multiple levels. AHE-supplemented protein diets attenuated obesity-associated metabolic dysfunction, improved longevity, and modulated metabolic signaling pathways without overt systemic toxicity [24,25,28,29]. More recent work demonstrated that AHE influenced hepatic β-catenin signaling, glutamine synthetase expression, amino acid metabolism, and renal inflammatory and ammonia-handling pathways [27]. Complementing these observations, the present study demonstrated that localized intratumoral NH_4_OH administration was not associated with overt renal toxicity and was accompanied by preserved renal morphology, reduced inflammatory and apoptotic signaling, and increased expression of the renal ammonia transporters RHBG and RHCG. Collectively, these studies established that controlled ammonium hydroxide exposure can modulate molecular pathways in multiple tissues while maintaining physiological homeostasis.

In this study, we were interested in determining whether a localized intratumoral injection of NH4OH would elicit systemic effects as well. Therefore, we selected renal tissue for further analysis based on (1) its importance in excreting ammonia-based metabolic waste [48] (2) the presence of known ammonia transporters [48,65] and (3) its functional impairment when exposed to chronic diseases (such as cancer) [66,67]. Here we expand this concept from potential dietary metabolic intervention to localized cancer therapy, demonstrating that direct intratumoral NH_4_OH administration selectively alters tumor biology while preserving renal tissue integrity.

Among the renal structures quantified, only the distal convoluted tubule (DCT) showed significant morphometric differences. The groups treated with NH showed no significant differences in DCT area and overall were slightly lower than the PBS control group, while differing significantly from the T-PBS group. This selective response may relate to the specialized roles of different nephron segments in ammonia handling and acid–base regulation. The proximal tubule is the major site of glutamine-dependent ammoniagenesis, the loop of Henle participates in ammonia recycling, and distal nephron segments, including the DCT and collecting duct, contribute to final urinary acidification through H^+^ secretion and ammonia trapping [47,48,65]. Tumors, including breast cancers, often generate an acidic microenvironment through glycolytic metabolism, lactate production, hypoxia, and impaired perfusion, and tumor-associated inflammatory and metabolic stress may influence systemic physiology [68,69]. Therefore, the increased DCT area observed in T-PBS mice compared with PBS controls may represent an adaptive response to tumor-associated inflammatory or metabolic stress affecting distal tubular function [70,71]. In contrast, the decreased DCT area in NH_4_OH-treated groups, together with increased RHBG/RHCG expression and reduced inflammatory markers, suggests that NH_4_OH did not exacerbate distal tubular remodeling and may have supported adaptive ammonia handling. However, because serum electrolytes, urinary ammonia, renal function, and systemic acid base status were not measured, the functional significance of this DCT change remains speculative and requires further investigation.

Together, these findings suggest that the biological and systemic effects of ammonium hydroxide are highly context-dependent, being influenced by the route of administration, tissue type, and metabolic state of the target cells.

A major strength of this study is the evaluation of kidney tissue as a measure of systemic safety. Because the kidney is central to ammonia transport and acid-base regulation [47,48], renal toxicity would be a major concern following NH_4_OH exposure. However, intratumoral NH_4_OH did not increase renal inflammatory, apoptotic, or histological injury markers. Instead, kidney tissues from NH_4_OH-treated tumor-bearing mice showed reduced TNFα, IL-1β, IL-6, TLR4, and Caspase-3 expression, unchanged p53, maintained BCL2 expression, and preserved renal morphology. In addition, RHBG and RHCG expression increased, suggesting an adaptive renal ammonia-handling response rather than tissue injury. This contrast between tumor and kidney responses suggests that NH_4_OH induced stress-associated signaling in metabolically vulnerable tumor cells while preserving renal homeostasis. Collectively, these findings indicate that intratumoral NH_4_OH treatment does not induce renal inflammation and may attenuate tumor-associated systemic inflammatory responses, as evidenced by reduced renal TNFα, IL-1β, IL-6, and TLR4 expression. Although representative IHC images suggested reduced renal p53 immunoreactivity following NH_4_OH treatment, quantitative Western blot analysis did not demonstrate a significant difference in total renal p53 protein expression. Because p53 functions primarily as a cellular stress-response protein with roles in DNA repair, cell-cycle regulation, senescence, and apoptosis, the present data do not permit conclusions regarding the specific biological pathway affected. Additional studies examining downstream p53 targets would be required to determine whether NH_4_OH influences p53-mediated stress responses or apoptotic signaling in the kidney. While more study is needed, these finding may have important implications for improved treatment responses and reducing treatment side effects on the renal system.

One question that needs to be addressed is whether the ammonium ions or the alkalization of the tumor microenvironment are the major factors we report in the tumor response. Studies have shown that targeted alkalization of the tumor microenvironment does not directly induce caspase-3 activation or apoptosis [72] and that increasing pH of the tumor microenvironment can promote DNA synthesis and cell proliferation, resulting in increased cell number and corresponding tumor volume [73]. Our results align with these reports as we specifically observed increases in caspase-3 and p53 with concurrent reduction in expression of KI67 in the NH_4_OH-treated tumors. This may also explain the lack of difference in tumor volume between treated and untreated cohorts. However, we did not investigate the recently identified phenomenon of alkaliptosis [74,75]. While investigation of this type of cell death may elucidate the effects of increased pH on the TME, it should be acknowledged that tumor cell type will also be a factor. Other studies have shown that both the “buffering agent” used and the type of administration plays a factor. For example, systemic administration of sodium bicarbonate in a mouse breast cancer model showed tumor promotion [76], whereas Dowlati et al. (2020) described antitumor activity in a non-small cell lung carcinoma model with the use of antibody-conjugated urease systems to generate ammonium ions locally [76].

One of the unique observations of the study was that systemic changes occurred in the renal tissues and tubules with intratumoral administration of a small volume (20 µL) of dilute ammonium hydroxide. The increases in ammonia transporters RHBG and RHCG, the lack of renal inflammation in the treated mice, coupled with the decreased DCT diameter suggests the use of ammonium hydroxide is key as the alkalization agent. Nevertheless, the regulation of the extracellular and intracellular regulation of pH in the TME is undeniably complex and requires careful consideration when contemplating clinical applications. A recent review by Chen et al. masterfully describes these challenges and the need for further study [77].

### Study Limitations

While this pilot study generated unexpected and compelling results, several limitations should be acknowledged. (1) The in vivo study used a single MDA-MB-231 xenograft model in athymic nude mice, limiting evaluation of immune-mediated mechanisms. (2) This was a pilot study. As such, intratumoral pH, ammonia concentration, glutamine metabolism, mitochondrial function, oxidative stress, or NH_4_OH pharmacokinetics were not measured.

In addition, direct assessment of renal function and homeostasis—including serum creatinine, blood urea nitrogen, electrolyte concentrations, urinary ammonia excretion, urinary pH, and glomerular filtration rate—was not performed. Therefore, conclusions regarding renal safety are limited to the molecular and histological findings presented in this study. Thus, future studies will need to incorporate more targeted analyses of these processes. (3) The sample size was small, and all treated animals received the same stepwise NH_4_OH dose-escalation regimen rather than a single fixed concentration throughout the treatment period. Although this strategy was selected to improve local tolerability in the absence of an established intratumoral NH_4_OH dosing protocol, it does not permit conclusions regarding an in vivo dose–response relationship or identification of the optimal therapeutic dose. Future studies using larger cohorts, multiple fixed NH_4_OH concentrations, additional breast cancer models, immunocompetent systems, metabolomic profiling, and optimized dosing strategies will be necessary to define the mechanism and translational potential of this approach.

## 5. Conclusions

In conclusion, this study provides preliminary information regarding the safety of localized intratumoral NH_4_OH administration. In the present study, highly diluted NH_4_OH was administered by direct intratumoral injection using a cautious stepwise dose-escalation regimen. Under these experimental conditions, no treatment-related morbidity, mortality, or significant body weight loss was observed. Furthermore, molecular and histological analyses of the kidney revealed no overt evidence of treatment-associated injury. Furthermore, localized intratumoral NH_4_OH administration into mammary tumors suppresses inflammatory and proliferative signaling, promotes apoptotic marker expression, and induces histological changes consistent with tumor tissue disruption. Importantly, these antitumor-associated effects occurred without evidence of overt renal toxicity and were accompanied by preserved renal morphology and increased renal ammonia transporter expression. As an initial preclinical study, these findings should be interpreted considering the relatively small sample size, and further studies are needed to validate these observations and elucidate the molecular mechanisms underlying the antitumor effects of intratumoral NH_4_OH. These findings suggest that localized administration of diluted NH_4_OH was well tolerated under the conditions evaluated in this pilot study. Nevertheless, additional preclinical toxicology, pharmacokinetic, and dose-optimization studies, together with comprehensive assessment of organ function, will be necessary before clinical translation can be considered.

## Figures and Tables

**Table 1:** Summary of in vitro and in vivo doses calculated for NH4OH.

Experimental Setting	Reported Dose	Approximate Nominal Concentration
In vitro selected low dose	2.5 µM	2.5 µM
In vitro selected high dose	45 µM	45 µM
In vitro screening maximum	225 µM	225 µM
In vivo first dose	0.01%	2.85 mM
In vivo second dose	0.1%	28.5 mM
In vivo third dose	0.5%	142.5 mM

**Table 2:** List of primers for mRNA.

Gene	Forward	Reverse
*TLR4*	5′-TTG CAT CTG GCT GGG ACT CTG-3′	5′-TTC AGG GGG TTG AAG CTC AGA T-3′
*NFkB*	5′-CCT CCA CCC CGA CGT ATT GC-3′	5′-GCC AAG GCC TGG TTT GAG AT-3′
*TNFα*	5′-GAA CTC CAG GCG GTG TCT GT-3′	5′-CTG AGT GTG AGG GTC TGG GC-3′
*IL1β*	5′-ATG TCT TGC CCG TGG AGC TT-3′	5′-ATG GGT CAG ACA GCA CGA GG-3′
*RHBG*	5′-GCCGTGTATCAGCTCTTCGGAA-3′	5′-AACCTGGTCCTCGAAGCATTGG-3′
*RHCG*	5′-CAGTTCCTTCCACGGAGATGCC-3′	5′-AAGCGTGGCATTCTGGATGTGC-3′
*B-actin*	5′-ACA ACC TTC TTG CAG CTC CTC C-3′	5′-TGA CCC ATA CCC ACC ATC ACA-3′

Abbreviations: *TLR4*, toll-like receptor 4, *NFkB*, nuclear factor kapp-light-chain-enhancer of activated B cells; *TNF-α*, tumor necrosis factor-α; *IL1β*, interleukin 1β; *RHBG*, Rhesus blood group-associated glycoprotein B, *RHCG*, Rhesus blood group-associated glycoprotein C.

**Table 3:** Summary of antibody dilutions and conditions used in Western blot analysis.

Primary Antibody	Species	Dilution	Vendor	Secondary Antibody	Dilution	Vendor
NFkB	Rabbit polyclonal	1:1000	Cell Signaling Technology, Inc., Danvers, MA, USA	Goat anti-rabbit HRP	1:2000	Cell Signaling Technology, Inc., Danvers, MA, USA
TNFα	Rabbit polyclonal	1:1000	Abcam, Cambridge, MA, USA	Goat anti-rabbit HRP	1:2000	Cell Signaling Technology, Inc., Danvers, MA, USA
TLR4	Mouse Monoclonal	1:1000	Novus Biological, Littleton, CO, USA	Rabbit anti mouse HRP	1:10,000	Abcam, Cambridge, MA, USA
IL6	Mouse Monoclonal	1:1000	Invitrogen, Carlsbad, CA, USA	Rabbit anti mouse HRP	1:10,000	Abcam, Cambridge, MA, USA
Caspase 3	Rabbit polyclonal	1:1000	Bioss Antibodies, Woburn, MA, USA	Goat anti-rabbit HRP	1:2000	Cell Signaling Technology, Inc., Danvers, MA, USA
P53	Rabbit polyclonal	1:1000	Abcam, Cambridge, MA, USA	Goat anti-rabbit HRP	1:2000	Cell Signaling Technology, Inc., Danvers, MA, USA
BCL2	Rabbit Monoclonal	1:1000	Cell Signaling Technology, Inc., Danvers, MA, USA	Goat anti-rabbit HRP	1:2000	Cell Signaling Technology, Inc., Danvers, MA, USA
RHBG	Rabbit polyclonal	1:1000	Thermo Fisher Scientific, Inc., Waltham, MA, USA	Goat anti-rabbit HRP	1:2000	Cell Signaling Technology, Inc., Danvers, MA, USA
RHCG	Rabbit polyclonal	1:1000	Bioss Antibodies, Woburn, MA, USA	Goat anti-rabbit HRP	1:2000	Cell Signaling Technology, Inc., Danvers, MA, USA
B-actin	Mouse Monoclonal	1:2000	Millipore Sigma (Burlington, MA, USA)	Horse anti-mouse HRP	1:2000	Cell Signaling Technology, Inc., Danvers, MA, USA

Abbreviations: TLR4, toll-like receptor 4, NFkB, nuclear factor kappa-light-chain-enhancer of activated B cells; TNF-α, tumor necrosis factor-α; IL6, interleukin-6; Caspase-3, cysteine-aspartic acid protease-3; p53, tumor protein p53; BCL2, B-cell lymphoma 2; RHBG, Rhesus blood group-associated glycoprotein B; RHCG, Rhesus blood group-associated glycoprotein C; β-actin, beta-actin.

## Data Availability

The original contributions presented in this study are included in the article/Supplementary Materials. Further inquiries can be directed to the corresponding author.

## Supplementary Materials

The following supporting information can be downloaded at https://www.mdpi.com/article/10.3390/cimb48080803/s1.
