# Supplementary material for: Localized Intratumoral Ammonium Hydroxide Administration Demonstrates Changes in Tumor Architecture and Renal Response in a Murine Breast Cancer Xenograft Model
Source: Curr Issues Mol Biol. 2026 Aug 7;48(8):803. doi: 10.3390/cimb48080803 (PMC13511212; doi:10.3390/cimb48080803)
Supplement: Supplementary file 1 [file cimb-48-00803-s001.zip › cimb-4442947-supplementary.pdf]

Figure S1. QuPath Digital Pathology Workflow

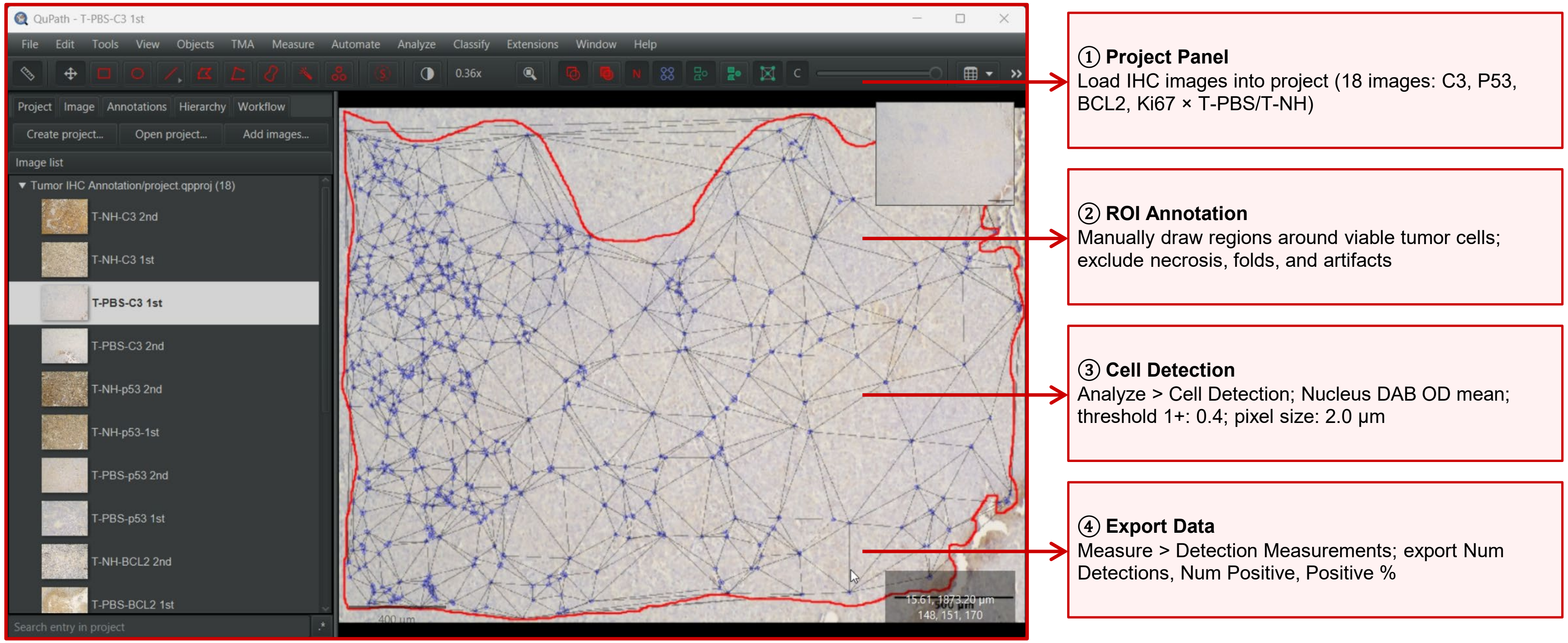

Red boundary = manually annotated ROI (viable tumor region). Blue dots = DAB-negative cells. Red dots = DAB-positive cells. Cell detection performed in QuPath using Nucleus DAB OD mean scoring (threshold 1+ = 0.4, pixel size = 2.0 μm).

Figure S2

A) IHC-Caspase 3

T-PBS-1 **T-PBS-2**

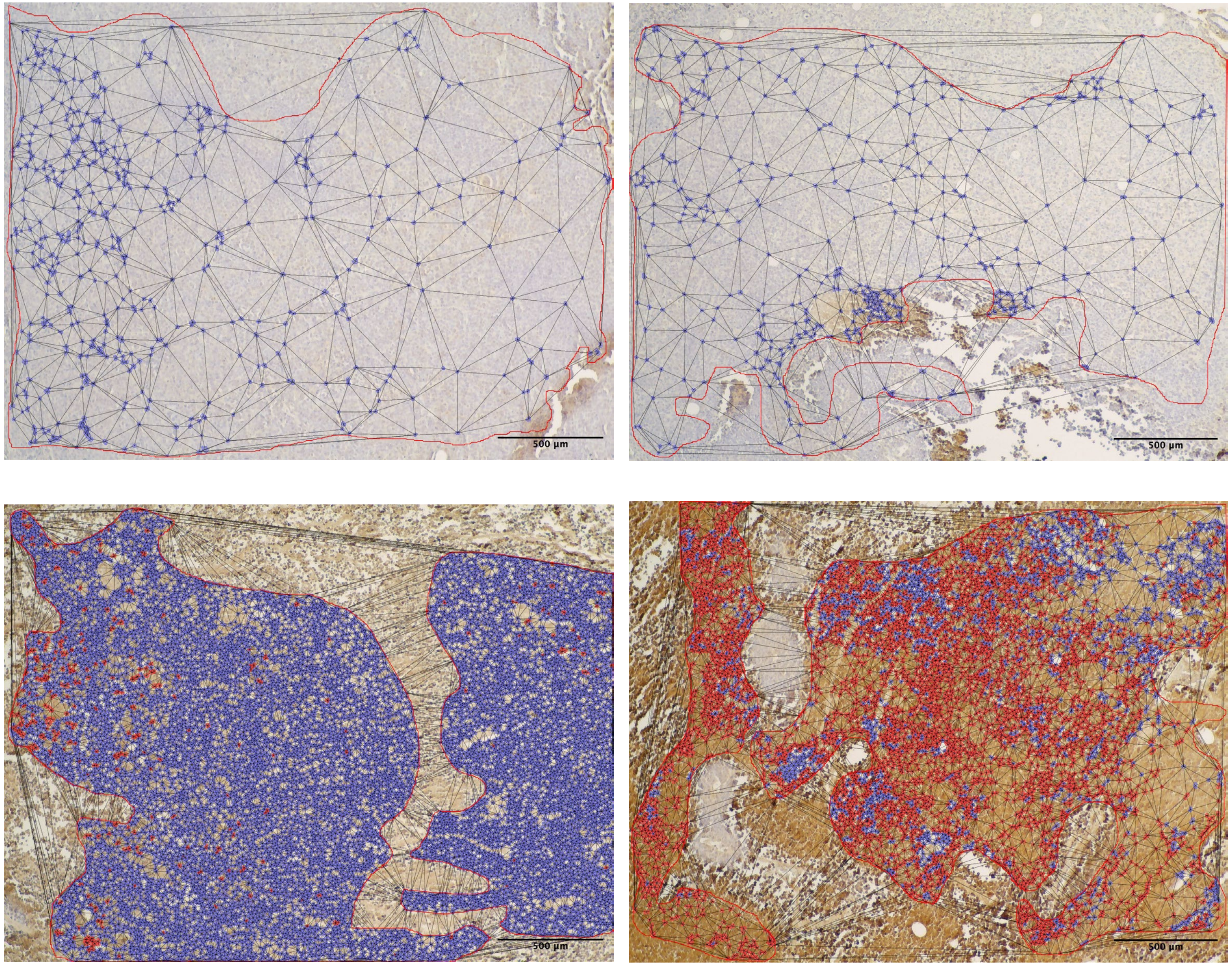

T-NH-1 **T-NH-2**

B) Raw Data

Sample	Annotation #	Num Detections	Num Negative	Num Positive	Positive %
T-PBS-C3 1st	1	533	533	0	0.00%
T-PBS-C3 2nd	1	479	479	0	0.00%
T-NH-C3 1st	1	8831	8518	313	3.54%
T-NH-C3 1st	2	4468	4432	36	0.81%
T-NH-C3 2nd	1	5269	1626	3643	69.14%
T-NH-C3 2nd	2	1117	163	954	85.41%

Figure S2: QuPath-annotated IHC images of Caspase 3 staining in T-PBS and T-NH tumor sections. Red dots indicate DAB-positive (Caspase 3-expressing) cells; blue dots indicate DAB-negative cells.
Quantification data generated by QuPath positive cell detection analysis.

Figure S3

A) IHC - p53

T-PBS-1 **T-PBS-2**

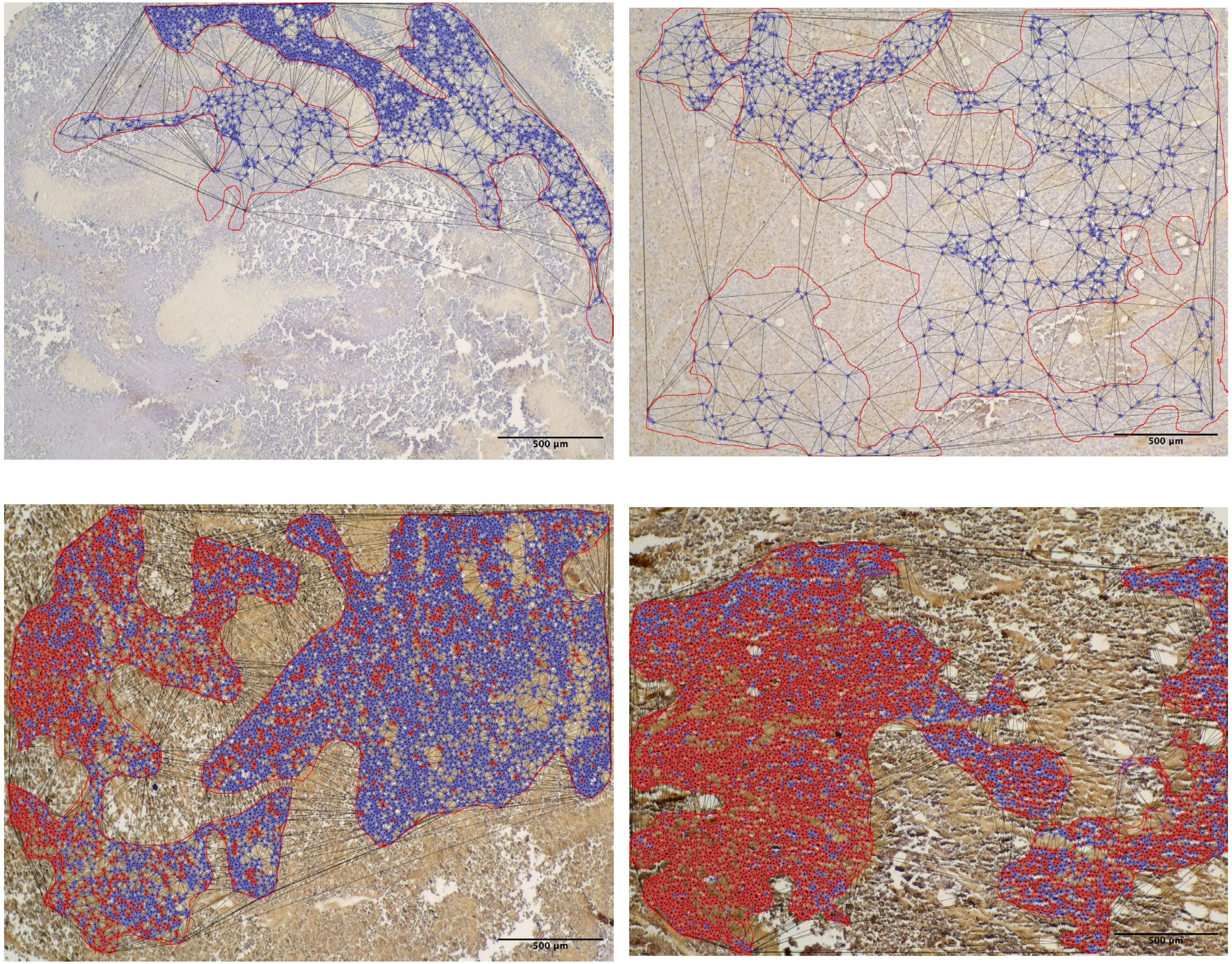

T-NH-1 **T-NH-2**

B) Raw Data

Sample	Annotation #	Num Detections	Num Negative	Num Positive	Positive %
T-PBS-p53 1st	1	1518	1518	0	0.00%
T-PBS-p53 2nd	1	315	315	0	0.00%
T-PBS-p53 2nd	2	68	68	0	0.00%
T-PBS-p53 2nd	3	437	437	0	0.00%
T-NH-p53 1st	1	5738	4700	1038	18.09%
T-NH-p53 1st	2	3735	1736	1999	53.52%
T-NH-p53 2nd	1	1674	639	1035	61.83%
T-NH-p53 2nd	2	6568	1460	5108	77.77%

Figure S3: QuPath-annotated IHC images of p53 staining in T-PBS and T-NH tumor sections. Red dots indicate DAB-positive (p53-expressing) cells; blue dots indicate DAB-negative cells. Quantification data generated by QuPath positive cell detection analysis.

Figure S4

A) IHC-BCL2

T-PBS-1 **T-PBS-2**

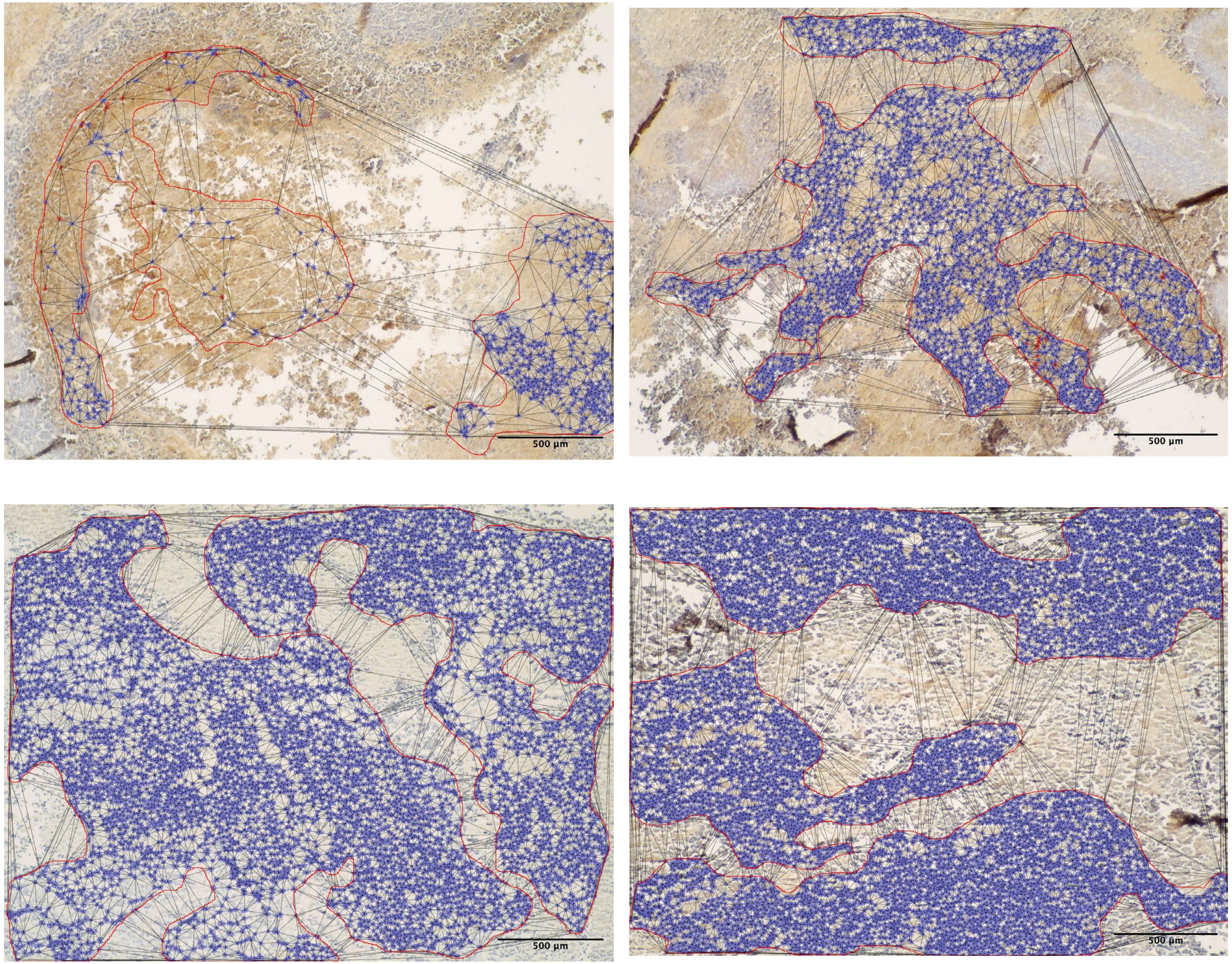

T-NH-1 **T-NH-2**

B) Raw Data

Sample	Annotation #	Num Detections	Num Negative	Num Positive	Positive %
T-PBS-BCL2 1st	1	171	155	16	9.36%
T-PBS-BCL2 1st	2	396	396	0	0.00%
T-PBS-BCL2 2nd	1	2680	2667	13	0.49%
T-NH-BCL2 1st	1	2733	2733	0	0.00%
T-NH-BCL2 1st	2	4448	4448	0	0.00%
T-NH-BCL2 2nd	1	3411	3410	1	0.03%
T-NH-BCL2 2nd	2	2990	2990	0	0.00%
T-NH-BCL2 2nd	3	1906	1906	0	0.00%

Figure S4: QuPath-annotated IHC images of BCL2 staining in T-PBS and T-NH tumor sections. Red dots indicate DAB-positive (BCL2-expressing) cells; blue dots indicate DAB-negative cells. Quantification data generated by QuPath positive cell detection analysis.

Figure S5

A) IHC-Ki67

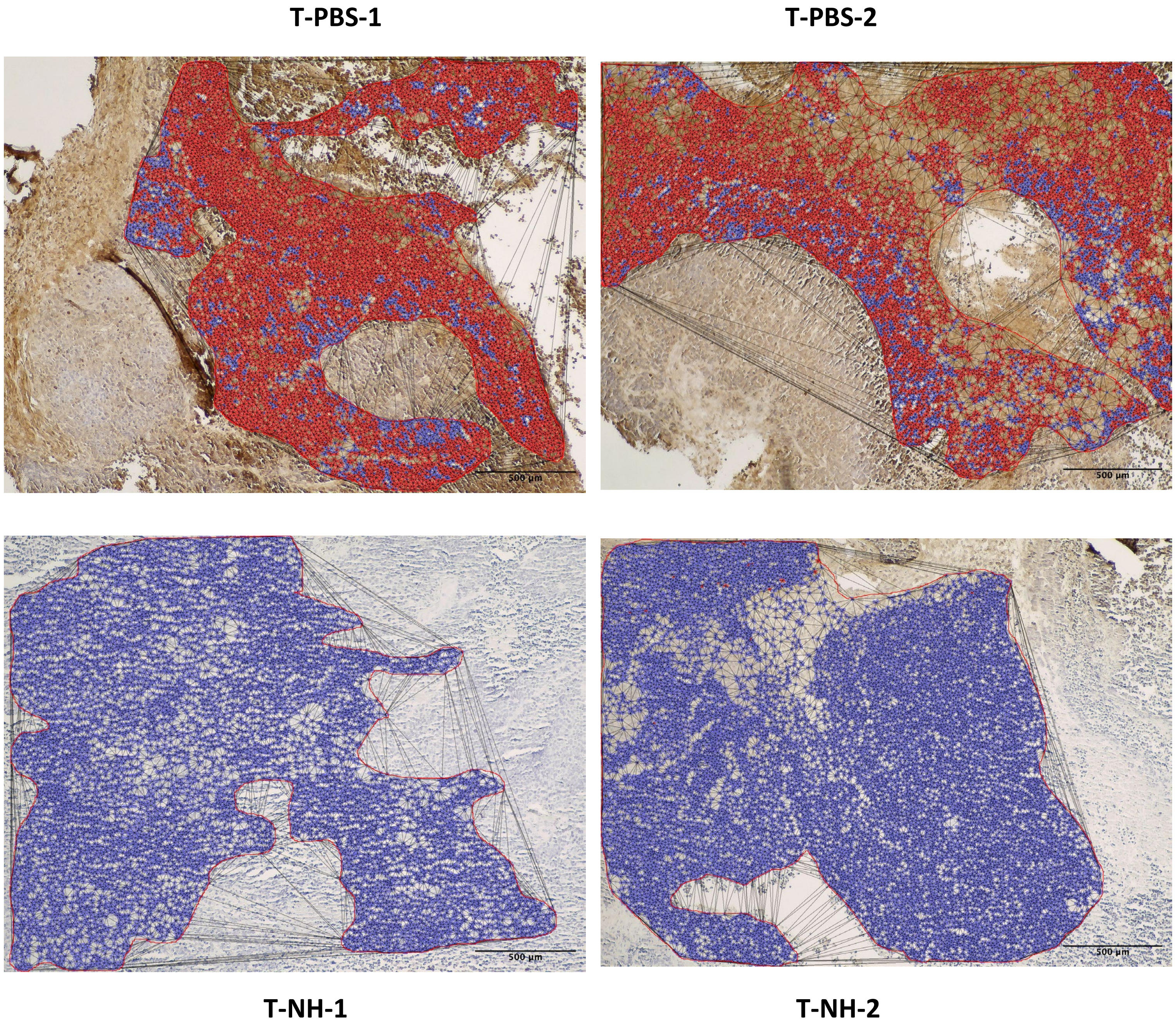

B) Raw Data

Sample	Annotation #	Num Detections	Num Negative	Num Positive	Positive %
T-PBS-Ki67 1st	1	8839	1680	7159	80.99%
T-PBS-Ki67 2nd	1	10261	2616	7645	74.51%
T-NH-Ki67 1st	1	9252	9252	0	0.00%
T-NH-Ki67 2nd	1	13895	13876	19	0.14%

Figure S5: QuPath-annotated IHC images of Ki67 staining in T-PBS and T-NH tumor sections. Red dots indicate DAB-positive (Ki67-expressing) cells; blue dots indicate DAB-negative cells. Quantification data generated by QuPath positive cell detection analysis.

Figure S6

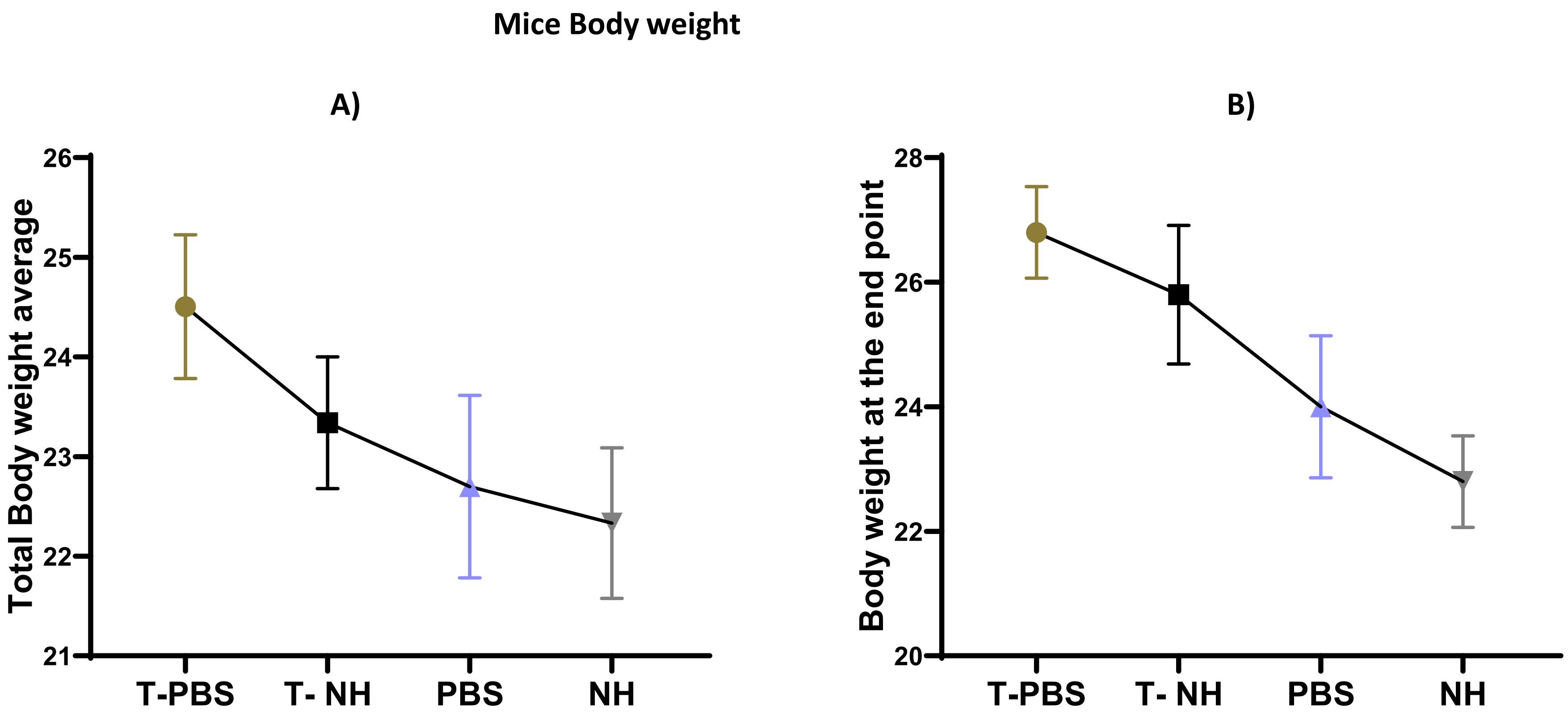

Supplementary Figure S6: Total average and end point body weight of mice following intratumoral NH_4OH treatment. Body weight was recorded A) twice weekly as well as at the B) experimental endpoint for T-PBS, T-NH, PBS control, and NH control mice. No significant differences were observed among the experimental groups. Data are presented as mean ± SEM. Statistical significance was determined with one-way ANOVA followed by Fisher's least significant difference (LSD) multiple comparisons test.

Figure S7

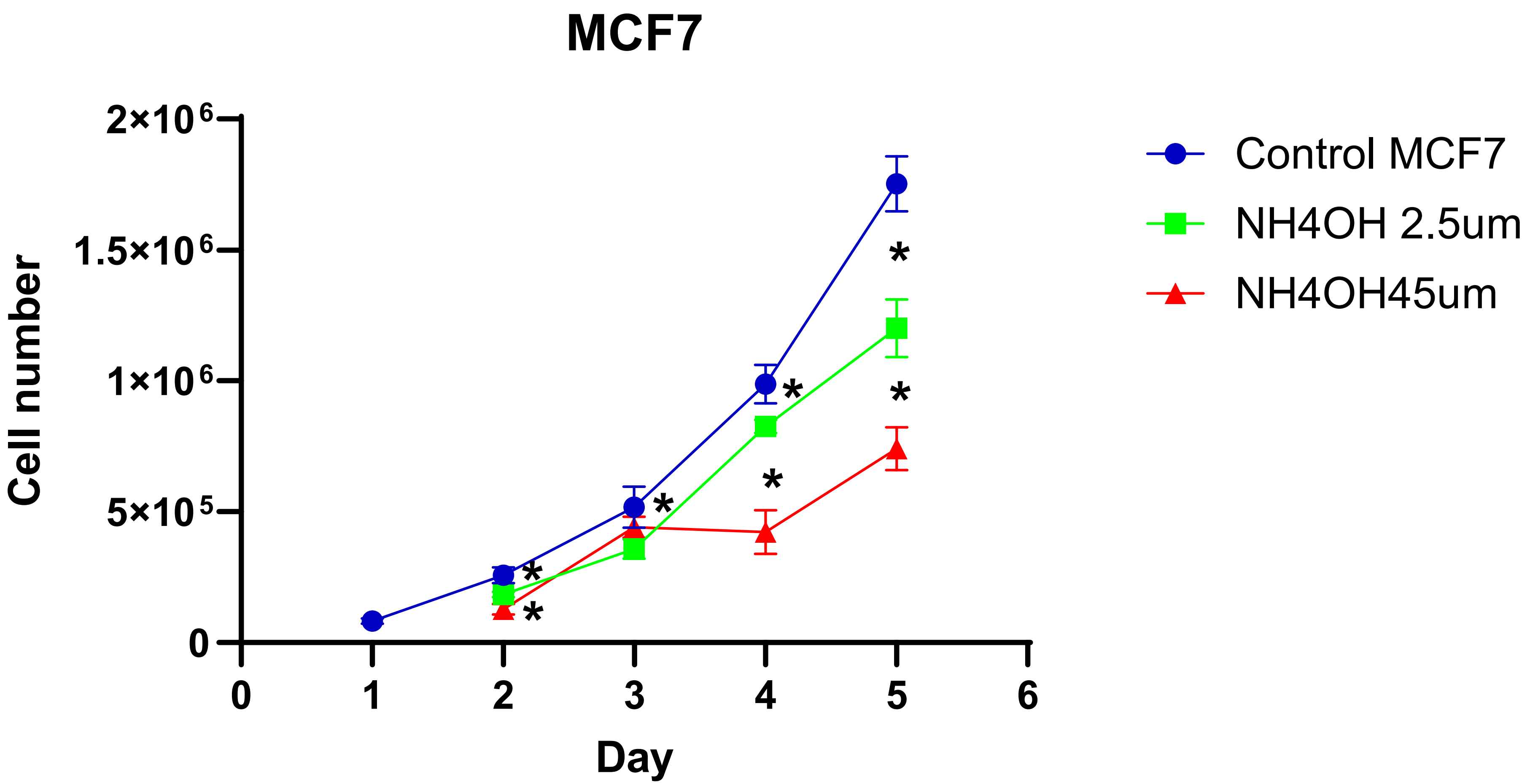

Supplementary Figure S7: Effects of NH_4OH on MCF-7 breast cancer cell viability. Representative growth curve of MCF-7 cells following NH_4OH treatment. Data are presented as mean ± SEM. Statistical significance was determined by one-way ANOVA followed by Fisher's least significant difference (LSD) multiple comparisons test.

Figure S8

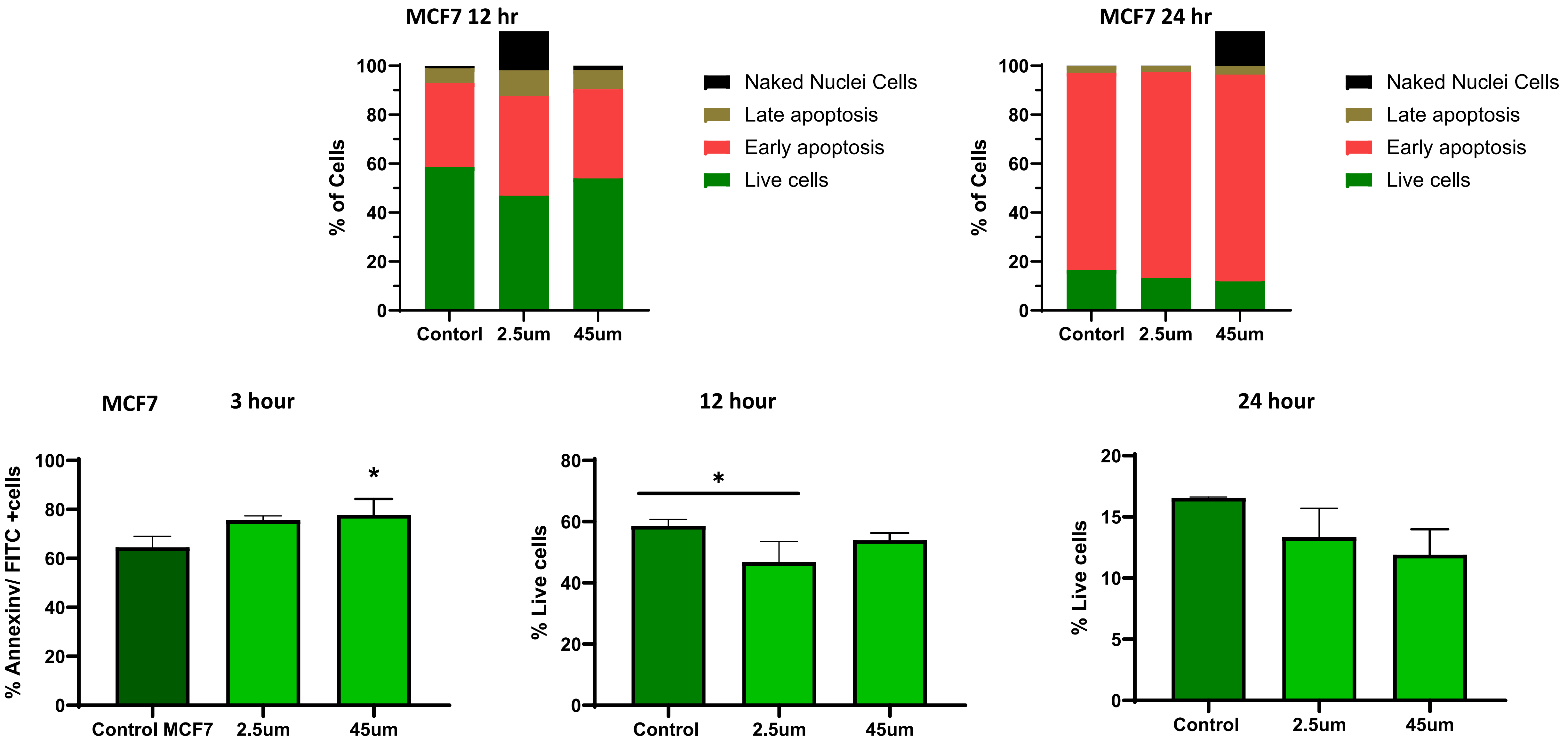

Supplementary Figure S8. Annexin V-FITC/PI analysis of NH_4OH-treated MCF-7 cells. Representative stacked bar graphs show the percentages of live cells, early apoptotic cells, late apoptotic cells, and naked nuclei in MCF-7 cells following treatment with NH_4OH (2.5 or 45 µM) for 12 h and 24 h. Quantification of the percentage of live cells after 3 h, 12 h, and 24 h of treatment is shown in the lower panels. Data are presented as mean ± SEM from three independent experiments. Statistical significance was determined using one-way ANOVA followed by Fisher's least significant difference (LSD) multiple-comparisons test. $P < 0.05$ compared with the untreated control.

Figure S9

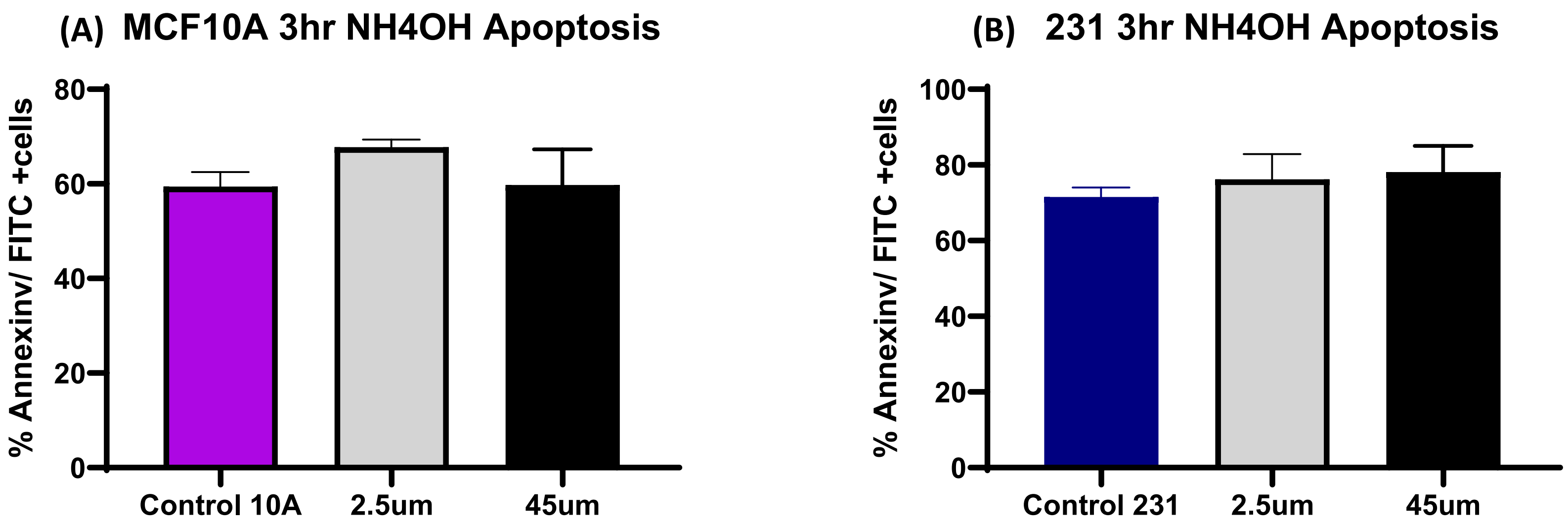

Supplementary Figure S9: Effects of ammonium hydroxide (NH_4OH) on apoptosis in MDA-MB-231 and MCF10A cells. Quantitative analysis of Annexin V-FITC/propidium iodide (PI) flow cytometry was performed to assess apoptosis in MCF10A(A) and MDA-MB-231(B) cells following treatment with NH_4OH at 0, 2.5 and 45µM for 3 h. Apoptotic cells were quantified as the percentage of Annexin V-FITC positive cells. Data are presented as mean ± SEM. Statistical significance was determined using one-way ANOVA followed by Fisher's least significant difference (LSD) multiple comparisons test. $P < 0.05$ was considered statistically significant.

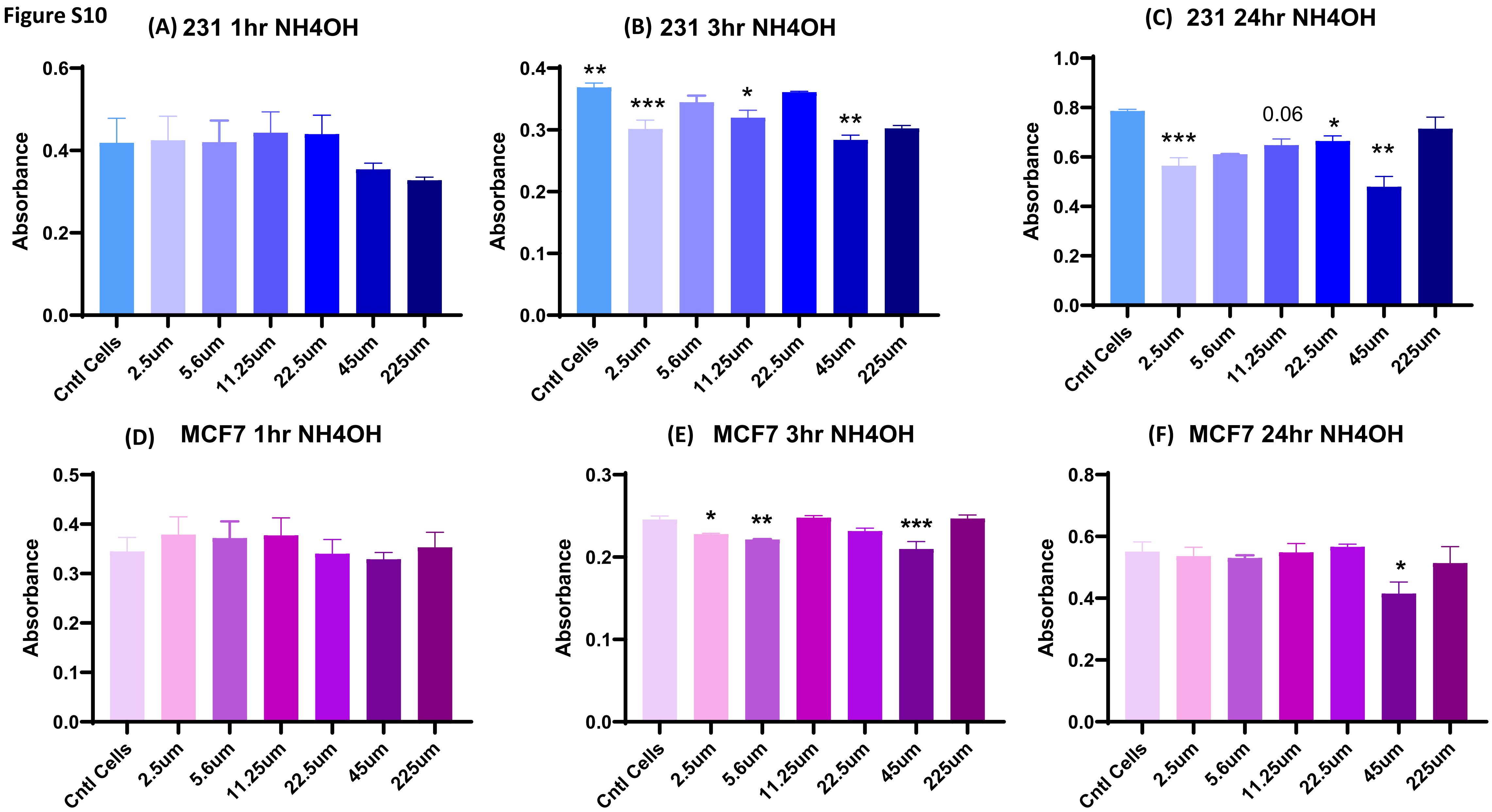

Supplementary Figure S10: Effects of ammonium hydroxide (NH_4OH) on cell viability in MDA-MB-231 and MCF-7 breast cancer cells assessed by XTT assay. MDA-MB-231 (A–C) and MCF-7 (D–F) cells were treated with increasing concentrations of NH_4OH and cell viability was determined using the XTT assay after 1 h (A, D), 3 h (B, E), and 24 h (C, F) of treatment. Data are presented as mean ± SEM. Statistical significance was determined using one-way ANOVA followed by Fisher's least significant difference (LSD) multiple comparisons test. $P < 0.05$ was considered statistically significant.

Figure S11

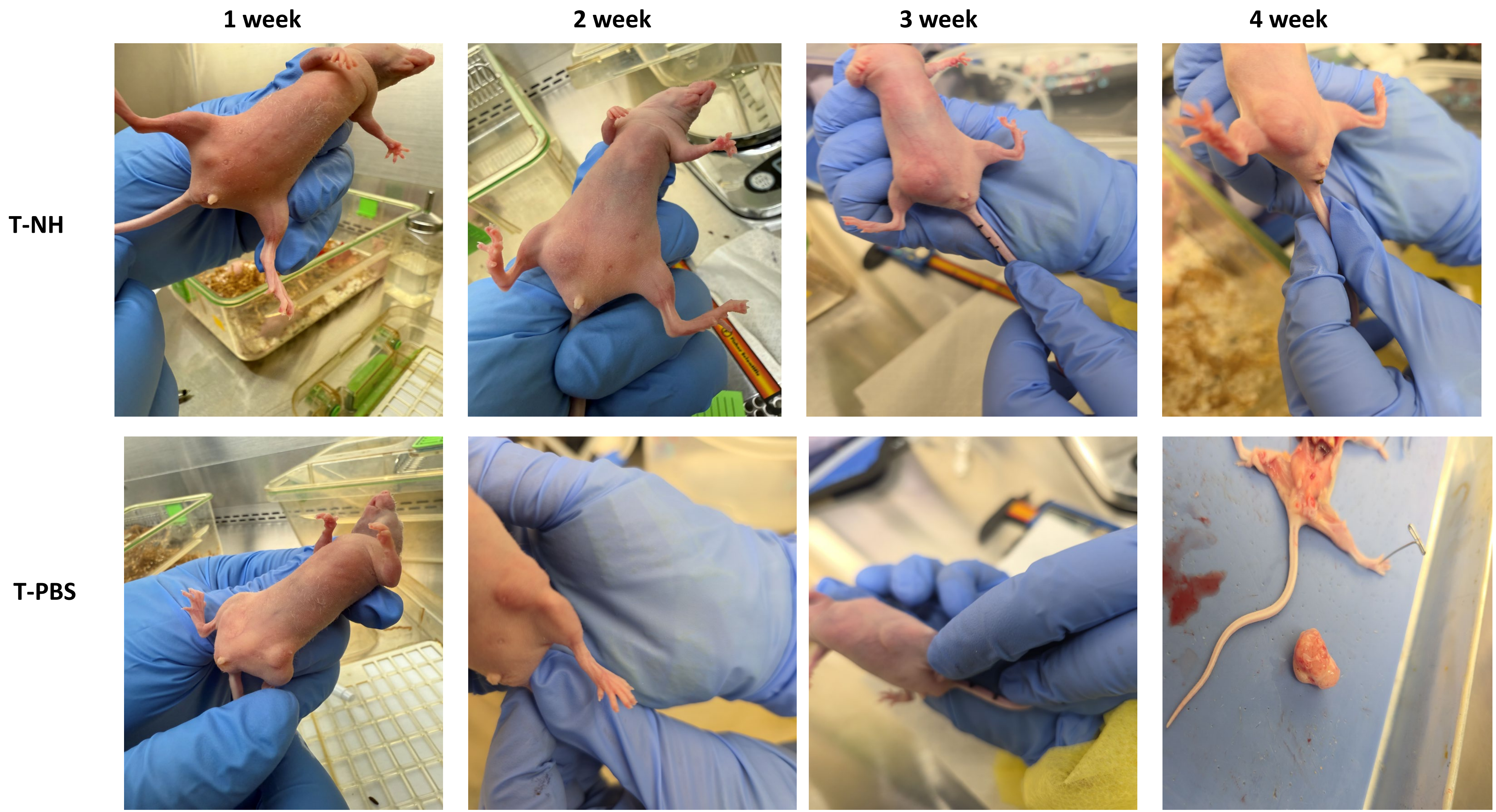

Supplementary Pictures S11. Images of the tumors during the study periods (per week after tumor growth was first observed).

Supplementary Table S1

MCF10A 12 hours

Treatment	Live Cells (%)	Early Apoptosis (%)	Late Apoptosis (%)	Naked Nuclei (%)
**Control**	83.8, 78.5, 73.8	9.9, 10.9, 14.2	2.2, 3.8, 2.5	4.0, 6.8, 0.5
**NH_4OH (2.5 µM)**	69.8, 80.3, 83.7	18.1, 7.1, 7.0	3.3, 2.0, 2.0	8.8, 10.7, 7.4
**NH_4OH (45 µM)**	79.5, 72.5, 71.5	12.2, 11.2, 14.6	2.5, 2.9, 3.2	5.8, 13.4, 10.7

MCF10A 24 hours

Treatment	Live Cells (%)	Early Apoptosis (%)	Late Apoptosis (%)	Naked Nuclei (%)
**Control**	36.4, 33.0, 33.3	54.9, 57.9, 57.8	7.5, 8.0, 8.2	1.2, 0.5, 0.7
**NH_4OH (2.5 µM)**	26.1, 27.8, 25.6	60.0, 57.0, 61.8	12.8, 13.9, 11.7	1.1, 1.3, 0.9
**NH_4OH (45 µM)**	31.5, 29.6, 41.7	56.2, 57.1, 43.9	11.6, 12.2, 12.7	0.6, 1.2, 1.7

Supplementary Table S1. Raw Annexin V-FITC/PI apoptosis data for MCF-10A cells following 12 h and 24 h NH_4OH treatment.
